# Endothelial PRMT7 prevents dysfunction, promotes revascularization and enhances cardiac recovery post-myocardial infarction

**DOI:** 10.1038/s12276-025-01517-x

**Published:** 2025-08-05

**Authors:** Thi Thuy Vy Tran, Yan Zhang, Shibo Wei, Jinwoo Lee, Yideul Jeong, Tuan Anh Vuong, Sang-Jin Lee, Dongryeol Ryu, Gyu-Un Bae, Jong-Sun Kang

**Affiliations:** 1 Department of Molecular Cell Biology, Sungkyunkwan University School of Medicine, Suwon, Republic of Korea; 2 Department of Biomedical Science and Engineering, Gwangju Institute of Science and Technology, Gwangju, Republic of Korea; 3 Research Institute of Aging-Related Diseases, AniMusCure Inc., Suwon, Republic of Korea; 4 Drug Information Research Institute, Muscle Physiome Research Center, College of Pharmacy, Sookmyung Women’s University, Seoul, Republic of Korea

**Keywords:** Cardiovascular diseases, Genetics research

## Abstract

Myocardial infarction (MI) induces ischemic damage, triggering endothelial cell (EC) dysfunction that impairs revascularization and cardiac recovery. A key contributor to this dysfunction is excessive endoplasmic reticulum (ER) stress, which is activated by MI and exacerbates EC apoptosis and impaired angiogenesis. Here we investigate the role of endothelial-specific protein arginine methyltransferase 7 (PRMT7) in mitigating ER stress and promoting vascular homeostasis after MI. We demonstrate that PRMT7 expression is upregulated in ECs under tumor necrosis factor α or tunicamycin treatment, while its inhibition exacerbates ER stress and induces EC death. Using endothelial-specific PRMT7-knockout models, we show that PRMT7 deficiency increases apoptosis and fibrosis, impairing cardiac recovery. Transcriptomic analysis reveals that PRMT7 loss leads to the upregulation of pro-apoptotic pathways and suppression of angiogenic and proliferative signaling. Conversely, PRMT7 overexpression or treatment with the PRMT7-inducing drug bindarit restores EC function, suppresses ER stress and enhances revascularization and cardiac repair after MI. These findings establish endothelial PRMT7 as a critical regulator of EC survival and function, highlighting its potential as a therapeutic target to mitigate ER stress and improve post-MI cardiac recovery.

## PRMT7 enhances endothelial function after myocardial infarction

Myocardial infarction (MI), commonly known as a heart attack, is a leading cause of death worldwide. Researchers are exploring ways to improve heart recovery after MI. This study focuses on a protein called PRMT7, which may help heart cells recover after a heart attack. Researchers used mice to study how endothelial PRMT7 affects heart recovery. They found that PRMT7 helps protect endothelial cells from stress and promotes the growth of new blood vessels, which is crucial for healing. They also discovered that a drug called bindarit can increase PRMT7 levels, improving heart recovery in mice after MI. The results suggest that PRMT7 plays a vital role in heart healing by reducing cell stress and promoting blood vessel growth. The study concludes that targeting endothelial PRMT7 could be a promising strategy for treating heart attacks.

This summary was initially drafted using artificial intelligence, then revised and fact-checked by the author.

## Introduction

Myocardial infarction (MI), a major cause of mortality worldwide, occurs due to coronary artery plaque rupture, leading to acute inflammation and myocardial necrosis^1,2^. Endothelial cells (ECs) play a crucial role in cardiac recovery by promoting revascularization and restoring perfusion to the infarcted area^3^. Early revascularization is essential for minimizing infarct size and limiting cardiac damage, with EC proliferation and migration being key processes in neovascularization and repair^4–6^. Consequently, identifying molecular regulators that control EC function is critical for developing therapeutic strategies to enhance cardiac recovery.

Inflammation and endoplasmic reticulum (ER) stress are major factors influencing MI outcomes^7,8^. While controlled inflammation is necessary for initiating tissue repair, excessive inflammation can induce apoptotic pathways, impairing cardiac recovery^9,10^. MI-induced ischemia disrupts ER homeostasis, triggering the unfolded protein response to restore balance^11,12^. However, prolonged ER stress can shift the response from adaptive to pro-apoptotic, mediated by CHOP activation, leading to endothelial dysfunction and cell death^13,14^. While transient ER stress promotes angiogenesis through AKT phosphorylation^15^, the precise molecular mechanisms that regulate the balance between EC survival and death after MI remain unclear.

Protein arginine methyltransferases (PRMTs) are epigenetic regulators that mediate the arginine methylation of histone and nonhistone proteins, influencing gene expression, cell signaling and stress responses^16,17^. PRMT family members have been implicated in cardiovascular function and pathology^18–22^. PRMT1 is the predominant asymmetric dimethyltransferase and is essential for maintenance of cardiac and vascular smooth muscle function^23,24^. PRMT5, a symmetric dimethyltransferase, regulates vascular development and angiogenesis after ischemic injury^25,26^. PRMT4/CARM1 and PRMT7 have been linked to stress responses and inflammatory pathways^27–30^. Our previous work demonstrated that PRMT7 deletion in cardiomyocytes exacerbates hypertrophy and fibrosis via dysregulated β-catenin signaling^31^. Given PRMT7’s role in modifying key signaling proteins, including β-catenin, p38 MAPK, HSP70 and eIF2α^32–35^, we hypothesized that PRMT7 is critical for EC function and cardiac recovery after MI.

Our study reveals that PRMT7 expression in ECs is upregulated by tumor necrosis factor α (TNF-α) and tunicamycin (TN), suggesting a stress-responsive role, whereas its inhibition exacerbates ER stress and induces EC death. Using an inducible EC-specific PRMT7-knockout model, we show that PRMT7 deficiency impairs cardiac recovery after MI, increasing apoptosis and fibrosis. Transcriptomic analysis revealed that endothelial PRMT7 loss upregulates pro-apoptotic pathways while suppressing angiogenic and proliferative signaling. Conversely, PRMT7 overexpression or treatment with the PRMT7-inducing drug bindarit restored EC function, suppressed ER stress and enhanced revascularization and cardiac repair after MI.

These findings identify endothelial PRMT7 as a key regulator of vascular homeostasis by modulating ER stress responses and apoptosis, highlighting its potential as a therapeutic target for improving EC survival and cardiac recovery after MI.

## Materials and methods

### Mouse experiments

*Prmt7*^tm1a(EUCOMM)Wtsi^ mice purchased from Sanger Institute were backcrossed onto C57BL/6J background for at least ten generations^36^. For the generation of inducible EC-specific PRMT7 null mice, *Prmt7*^Tm1c/Tm1c^ (*Prmt7*^f/f^) mice were crossed with mice carrying *Cdh5*-CreERT2 transgene (Jackson Laboratory; Tg(Cdh5-cre)7Mlia/J). To assess the role of endothelial PRMT7 in cardiovascular disease, these studies used about 3-month-old littermate male mice from heterozygous breeding. To induce the ablation of PRMT7 in ECs, *Prmt7*^fl/fl; Cdh5-ERT2Cre^ mice were treated with tamoxifen for 2 weeks^24^.

MI mouse models were established by left anterior descending (LAD) coronary artery ligation without mechanical ventilation, as previously reported^37^, and LAD ligation was performed on 10-week-old mice. In brief, mice were subjected to anesthesia using 2% isoflurane inhalation without ventilation. A skin incision of 0.5 cm was made over the left chest, and a purse suture was created using 4–0 nonabsorbable Prolene sutures. A small opening was created by a mosquito clamp in the fourth intercostal space exposed by blunt dissection of the pectoral muscles. The heart was then carefully popped out from the pleura and pericardium by slightly opening the clamp and pressurizing the right thorax. The LAD was located and ligated using 7–0 Prolene sutures. After ligation, the heart was immediately repositioned back into the intrathoracic space, followed by manual evacuation of air and closure of the purse and skin. The mice were placed on an insulation mat and allowed to breathe room air, generally regaining consciousness within 3 min. The sham group underwent the same surgical procedure without the occlusion of the LAD. The animal studies were approved by the Institutional Animal Care and Use Committee of Sungkyunkwan University School of Medicine and conducted in accordance with the ethical guidelines (protocol number SKKUIACUC2023-02-21-1).

### Echocardiography analysis

Mice were anesthetized with 1% (vol/vol) isoflurane. Echocardiography was performed on mice 1 and 3 weeks after MI, using a Vevo LAZR-X photoacoustic imaging system (FUJIFILM VisualSonics). Heart rates (HRs) were monitored and generally maintained at 400–500 beats per minute. Analyses of M-mode images derived from the short-axis view of the left ventricle (LV) were performed to calculate the ejection fraction (EF) and fractional shortening (FS).

### Histology and immunofluorescence

Histology of heart sections was performed as previously described^38^. In brief, collected mouse hearts were fixed with 4% paraformaldehyde and embedded into paraffin block or Tissue-Tek optimal cutting temperature compound (Sakura Finetec). Paraffin-embedded or frozen heart tissues were sectioned at 7 µm thickness and subjected to histological analysis, such as hematoxylin and eosin (H&E, BBC Biochemical) and Masson’s trichrome (Abcam). The images were captured using a Nikon ECLIPS TE-2000U inverted microscope and Tissue FAXS imaging software (TissueGnostics). In addition, immunofluorescence staining of heart samples was performed as described previously^39^. In brief, heart sections were fixed using 4% paraformaldehyde and subsequently permeabilized with 0.5% Triton X-100 to facilitate antibody penetration. Antigen retrieval was achieved by immersing the sections in a sodium citrate solution at pH 6.0, heated to boiling for 15 min. Blocking was accomplished with a 5% solution of goat serum. Subsequent immunostaining involved sequential incubation with primary antibodies as outlined in Supplementary Table 1. Fluorescent images were obtained by using LSM-710 confocal microscope system (Carl Zeiss) or Cytation C10 confocal microscope system (Agilent). To quantify the signal intensity, images were analyzed with ImageJ software.

### Evans blue staining

To assess cell damage in myocardium, Evans blue staining was utilized following a previously established protocol^40^. In brief, Evans blue (Sigma-Aldrich) was dissolved in phosphate-buffered saline (PBS, 10 mg/ml) and sterilized by passage through membrane filters with a pore size of 0.2 μm. The dye solution was injected into mice from the tail vein at a final concentration of 1% (w/v). Three hours after injection, mice were euthanized and visually inspected for dye uptake into myocardium, indicated by blue coloration.

### TUNEL assay

To investigate cell apoptosis in myocardium, terminal deoxynucleotidyl transferase dUTP nick-end labeling (TUNEL) imaging assays (Click-iT Plus TUNEL Assay) were performed following the manufacturer’s instructions. In brief, heart sections were permeabilized, and a mixture of terminal deoxynucleotidyl transferase reaction buffer and EdUTP were applied to the sections. After 60 min incubation, sections were rinsed with 3% bovine serum albumin (BSA) and 0.1% Triton X-100 in PBS for 5 min and treated with TUNEL reaction cocktail mixture for 30 min, then mounted with mounting solution with 4′,6-diamidino-2-phenylindole (DAPI, Abcam) to visualize nuclei. Images were analyzed with the Cytation C10 confocal microscope system (Agilent).

### Cell cultures

C166 cells were cultured in Dulbecco’s modified Eagle medium (Gibco) containing 10% fetal bovine serum (Gibco) and 1% penicillin–streptomycin under standard culture conditions (37 °C and 5% CO_2_). To assess the effects of PRMT7 inhibition under stress, cells were treated with 1 µM SGC8158, an inhibitor for PRMT7 (Sigma-Aldrich) in combination with 0.1% BSA or 50 ng/ml TNF-α (PeproTech). To promote PRMT7 overexpression, C166 cells were transfected with either pcDNA3.1 vector or pcDNA3.1 carrying *Prmt7*-HA for 8 h using polyethylenimine (1 mg/ml, Sigma-Aldrich) as the transfection agent. After that, the transfection medium was replaced with fresh medium, and cells were cultured for an additional 24 h before subsequent experimental procedures.

### Bromodeoxyuridine (BrdU) staining

To examine the proliferation property, C166 cells were allowed to grow on chamber slides at proper confluency. After treatment with different conditions, cells were cultured with fresh media containing BrdU at 10 μM for 1 h and fixed with ice-cold methanol for 4 min at 4 °C, followed by hydrochloride denaturation at 2 M for 20 min at room temperature. Cells were blocked with 3% BSA in PBS for 1 hour at room temperature and treated with primary anti-BrdU antibody and Alexa Fluor 546 secondary antibody, followed by mounting using mounting solution with DAPI (Abcam). Images were analyzed with the Cytation-C10 confocal microscope system (Agilent).

### Scratch assay

To examine the migratory capability, 5 × 10^4^ C166 cells were seeded in six-well plates and allowed to grow until reaching a confluency of approximately 90%. A mechanical scratch was created on the cell monolayer using a pipette tip. Images were then captured after incubation for 0 or 24 h, and cell migration was quantified as the percentage of wound closure.

### Tube formation assay

To assess the angiogenic potential of ECs in vitro, 5 × 10^4^ C166 cells were seeded onto Matrigel-coated 24-well plates and allowed to form capillary-like structures for a duration of 2 h.

### Protein analysis

Immunoblotting analysis was performed as previous described^41^. In brief, heart tissues or cultured cells were lysed with lysis buffer composed of 10 mM Tris–HCl (pH 8.0), 150 mM NaCl, 1 mM EDTA, 1% Triton X-100 and complete protease inhibitor cocktail (Roche Diagnostics). The protein concentration was measured spectrophotometrically at 562 nm using the BCA protein assay reagent (Pierce). Cell lysates were subjected to SDS–PAGE (Bio-Rad) and transferred to polyvinylidene difluoride membranes (Bio-Rad). The membranes were blocked with 5% bovine serum albumin in Tris-buffered saline (TBST) for 30 min and incubated with the indicated primary antibody overnight at 4 °C. The blots were washed with TBST for 20 min and then incubated with peroxidase-conjugated secondary antibody for 1 h at room temperature. Blots were washed with TBST for 20 min and visualized. Protein levels were quantified by analyzing signal intensity using the ImageJ (NIH) program and normalized to the loading controls. The primary antibodies used in this study are listed in Supplementary Table 1.

### RNA analysis

Quantitative real-time polymerase chain reaction (qRT-PCR) and total RNA sequencing analysis were performed as previously described^42^. Total RNAs from mouse hearts and C166 cells were extracted with easy-BLUE (iNtRON) reagent following the manufacturer’s instructions. The primer sequences for qRT-PCR are listed in Supplementary Table 2. cDNA samples were generated from 0.5 µg of RNAs with PrimeScript RT reagent kit (TaKaRa) according to the manufacturer’s protocol. Total RNA sequencing analysis of hearts was carried out with an Agilent 2100 bioanalyzer using the RNA 6000 Nano Chip (Agilent Technologies). RNA sequencing data analysis was performed using ExDEGA v1.61 (e-Biogen) and ClueGO/CluePedia, EnrichmentMap and GeneMania plugins from Cytoscape (v3.10.0) software. The global gene expression was assessed by biological process with gene set enrichment analysis using MSigDB database v6.1 (>1.3-fold, Read Count log_2_ >2, *P* < 0.05).

### scRNA-seq dataset analysis

Previously published single-cell RNA sequencing (scRNA-seq) data deposited in Gene Expression Omnibus under accession number GSE201947 (ref. ^43^), and published single-nucleus RNA sequencing data^44^ available via Zenodo at 10.5281/zenodo.6578046 (ref. ^45^), are used to further assess the role of PRMT7 in ECs. All scRNA-seq datasets were analyzed using the SeqGeqTM (three stars) program. The datasets were integrated using the pipeline of the Dimensionality Reduction platform. Integrated cells underwent a quality control process to remove dead cells and doublets, which was based on library size and cells expressing dispersion. Subsequently, the integrated cells were normalized as counts per million. The filtered cells were then clustered using the Seurat pipeline with a resolution of 0.5. To visualize the data, a dimensional reduction uniform manifold approximation and projection was generated through the Seurat function RunUMAP. Cluster identities were assigned on the basis of top marker genes and the expression of known marker genes from published literature. The differentially expressed genes in the scRNA-seq database were extracted on the basis of *P* < 0.05 and fold change >1.5. For principal component analysis (PCA) analysis, selected genes in each group from RNA sequencing samples were extracted on the basis of *P* < 0.05 and log_2_ expression >2.0 and automatically clustered with 20 variances using SeqGeq software.

### GO enrichment analysis of DEGs

The Cytoscape (v3.1.0) cluego plugin was used to visualize enriched pathways associated with biological pathway databases. In brief, biological Gene Ontology (GO) terms were explored with medium specificity and a kappa score of 0.4. An enrichment/depletion method with a two-sided hypergeometric test was applied with Bonferroni step down for each *P*-value calculation. Enriched pathways with a *P* value <0.05 were considered significant. Gene set enrichment analysis was performed to extract knowledge of overrepresented GO terms for various functional processes and signaling pathways between each sample. Visualization of significantly enriched GO terms of functional process and signaling pathways between samples was performed with the Cytoscape plugin EnrichmentMap. The mapping of gene expression levels was done using the GeneMania plugin. All GO terms in our network analysis were filtered on the basis of pathway scores, retaining only those with a *P* value below 0.05.

### Statistical analysis

Statistical significance was calculated using the two-tailed paired or unpaired Student’s *t*-test between two groups, with one-way analysis of variance (ANOVA) among three or more groups using GraphPad Prism 8.4.3 (GraphPad Software). *P < 0.05, **P < 0.01, ***P < 0.001 and ****P < 0.0001 were considered statistically significant.

## Results

### PRMT7 expression is elevated in ECs in response to cellular stress, and its inhibition aggravates stress-induced ER stress and promotes cell death

**Fig. 1:**
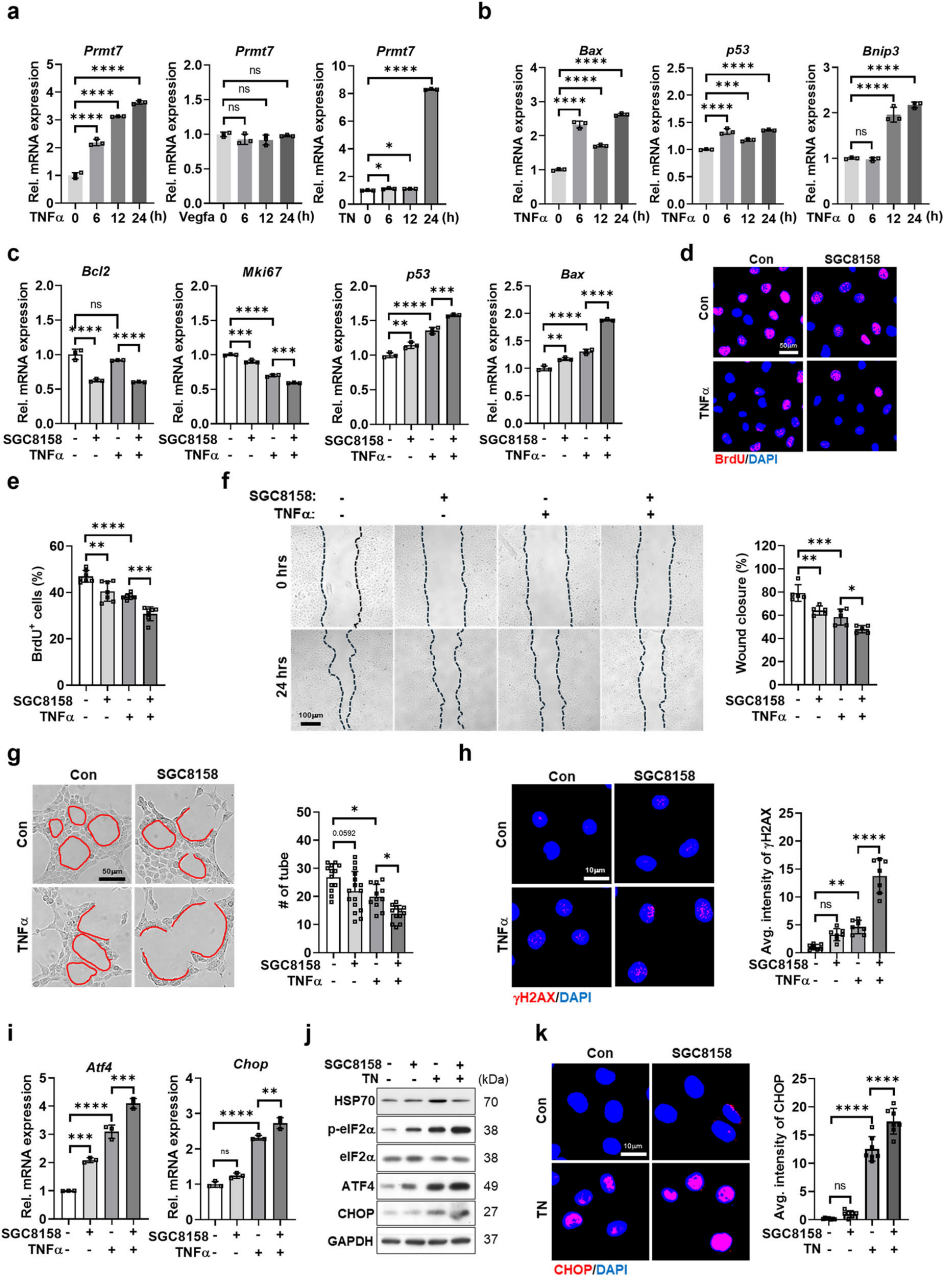
PRMT7 inhibition aggravates stress-induced ER stress and enhances cell death in C166 cells. **a** qRT-PCR analysis of *Prmt7* mRNA expression in C166 cells treated with VEGFA (100 ng/ml), TNF-α (50 ng/ml) and TN (10 µg/ml) for different time durations (0, 6, 12 and 24 h). **b** qRT-PCR analysis of *Bax*, *p53* and *Bnip3* mRNA expression in C166 cells treated with TNF-α (50 ng/ml) for different time durations (0, 6, 12 and 24 h). **c** qRT-PCR analysis of *Bcl2*, *Mki67*, *p53* and *Bax* mRNA expression in C166 cells treated with dimethyl sulfoxide (DMSO), SGC8158 (1 µM), TNF-α (50 ng/ml) or a combination of SGC8158 and TNF-α for 24 h. **d** Representative images of BrdU staining (red) and DAPI (blue) in C166 cells treated under the same conditions as in **c**. Scale bar, 50 µm. **e** Quantification of the percentage of BrdU-positive cells. **f** Representative images of the scratch wound healing assay in C166 cells treated under the same conditions as in **c** for 0 h or 24 h. Scale bar, 100 µm. Quantification of wound closure percentage from the scratch wound healing assay. **g** Representative images of the tube formation assay in C166 cells treated under the same conditions as in **c.** Scale bar, 50 µm. Quantification of tube number. **h** Representative images of immunostaining for DNA damage (γH2AX, red) and counterstained with DAPI (blue) in C166 cells treated under the same conditions as in **c**. Scale bar, 10 µm. Quantification of average intensity of γH2AX in C166 cells. **i** qRT-PCR analysis of *Atf4* and *Chop* mRNA expression in C166 cells. **j** Immunoblot analysis of HSP70, p-eIF2α, eIF2α, CHOP, ATF4 and loading control (GAPDH) protein levels in C166 cells treated with DMSO, SGC8158 (1 µM), TN (10 µg/ml) or a combination of SGC8158 and TN for 24 h. **k** Representative images of immunostaining for CHOP (red), and counterstained with DAPI (blue), in C166 cells treated with DMSO, SGC8158 (1 µM), TN (10 µg/ml) or a combination of SGC8158 and TN for 24 h. Scale bar, 10 µm. Quantification of the average intensity of CHOP. All data are presented as mean ± s.d. One-way ANOVA. ns, *P* > 0.05; *P < 0.05, **P < 0.01, ***P < 0.001, ****P < 0.0001.

To examine PRMT7’s role under stress, C166 ECs were treated with VEGFA, TN or TNF-α, followed by an assessment of *Prmt7* expression and stress-related markers. While VEGFA had no effect, TNF-α and TN significantly upregulated *Prmt7*, correlating with increased cell death regulators *p53*, *Bax* and *Bnip3*, suggesting the involvement of PRMT7 in cytotoxic stress responses (Fig. 1a, b and Supplementary Fig. 1a,b).

To further explore this, ECs were treated with the PRMT7 inhibitor SGC8158 (1 µM) alongside TNF-α or TN. PRMT7 inhibition reduced survival and proliferation markers (*Mki67* and *Bcl2*) and increased cell death regulators (*p53* and *Bax*) (Fig. 1c). BrdU incorporation assays confirmed a decline in proliferation following PRMT7 inhibition or TNF-α treatment, with a more pronounced reduction under combined treatment (Fig. 1d, e).

Functional assays revealed that PRMT7 inhibition impaired EC migration and angiogenesis. Scratch and wound healing assays showed significantly reduced wound closure with PRMT7 inhibition or TNF-α treatment, with an even greater effect when combined (Fig. 1f). Similarly, tube formation assays demonstrated that PRMT7 inhibition worsened the already reduced tube-forming capacity of TNF-α-treated ECs (Fig. 1g).

Further analysis indicated that PRMT7 inhibition enhanced cellular stress responses. γH2AX accumulation, a marker of DNA damage, was elevated in TNF-α- or TN-treated cells following PRMT7 inhibition (Fig. 1h and Supplementary Fig. 1c). PRMT7 inhibition also amplified TNF-α- and TN-induced ER stress, as indicated by increased expression of *Atf4* and *Chop* (Fig. 1i and Supplementary Fig. 1d). In addition, phosphorylated eIF2α (p-eIF2α), ATF4 and CHOP levels, as well as CHOP-positive cell numbers, were further elevated in co-treated ECs compared with controls and single-treatment groups (Fig. 1j, k and Supplementary Fig. 1e). A previous study reported that PRMT7 methylates HSP70 at arginine 469, thereby modulating the stress responses^34^. In our study, TN treatment elevated HSP70 protein levels, while pharmacological inhibition of PRMT7 attenuated this effect. These findings suggest a positive relationship between PRMT7 and HSP70 under stress conditions.

Collectively, these findings suggest that PRMT7 plays a critical role in protecting ECs under stress by modulating survival, proliferation, migration and the ER stress response.

### PRMT7 expression is elevated in heart tissues and ECs from patients with MI as well as in mouse MI heart samples

To examine the role of PRMT7 in MI, we analyzed published scRNA-seq database from heart samples from patients with MI^44^. While ventricular cardiomyocytes decreased, ECs, fibroblasts and immune cells increased (Supplementary Fig. 2a). *PRMT7* expression was elevated, especially in the ischemic zone, peaking in the acute phase and returning to baseline during fibrogenesis (Supplementary Fig. 2b). Among cell types, endothelial, cycling and immune cells exhibited increased *PRMT7* expression after MI (Supplementary Fig. 2c,d). *PRMT7* expression in ECs was categorized into three levels: negative, intermediate and high (Supplementary Fig. 2e). Gene set enrichment analysis revealed that ECs with high *PRMT7* expression were associated with innate response, regulation of cation channel activity, collagen fibril organization and plasminogen activation. By contrast, *PRMT7*-negative ECs were linked to pathways involving organelle assembly, muscle contraction. Notably, ECs with intermediate *PRMT7* expressions were enriched for pathways related to EC migration, cell cycle and proliferation. Similarly, the mouse MI database (GSE201947) showed heightened *Prmt7* levels in ECs 1 week after MI, aligning with human data (Supplementary Fig. 2f,g). Our MI mouse model confirmed PRMT7 upregulation in cardiomyocytes and microvascular ECs (Supplementary Fig. 2h,i). Three weeks after MI, *Prmt7* levels remained elevated alongside *Icam1* and pro-inflammatory cytokines (Supplementary Fig. 2j), suggesting PRMT7’s role in revascularization and cardiac recovery.

### EC-specific deletion of PRMT7 exacerbates MI-induced cardiac dysfunction

To investigate endothelial PRMT7’s role in MI, we generated a mouse model with inducible EC-specific PRMT7 deletion. *Prmt7*^fl/fl^ mice were crossed with *Cdh5*-Cre/ERT2 transgenic mice to create *Prmt7*^fl/fl^; *Cdh5*-ERT2Cre mice, where tamoxifen treatment induced EC-specific PRMT7 deletion (EndoKO). At 3 months, EndoKO and wild-type (WT) mice showed no significant cardiac differences, indicating that endothelial PRMT7 deletion alone does not impair baseline heart function (Supplementary Fig. 3a).

**Fig. 2:**
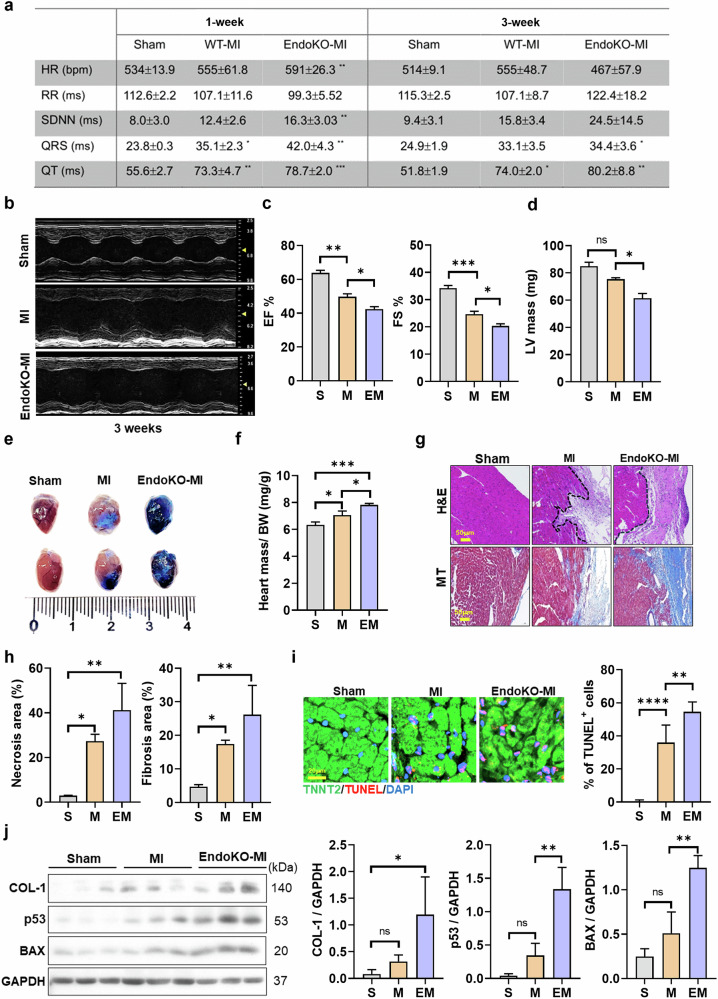
Endothelial PRMT7 ablation exacerbates MI-induced cardiac dysfunction. **a** Electrocardiography parameters of sham, MI and endothelial PRMT7-knockout (EndoKO)-MI mice 1 and 3 weeks after MI. **b** Representative echocardiographic images of sham (S), MI (M) and EndoKO-MI (EM) mice 3 weeks after MI. **c** EF and FS of S, MI and EM 3 weeks after MI. **d** LV mass 3 weeks after MI. **e** Representative images of hearts stained with Evans blue 3 weeks after MI. **f** Relative heart weight normalized by the body weight 3 weeks after MI. **g** H&E staining and Masson’s trichrome staining of hearts 3 weeks after MI. Scale bar, 50 µm. **h** Quantification of necrosis and fibrosis area of heart tissues. **i** Representative images of TUNEL (red), staining with cardiomyocyte marker (TNNT2, green) and counterstained with DAPI (blue). Scale bar, 20 µm. Quantification of the percentage of TUNEL-positive cells. **j** Immunoblot analysis of collagen type I (COL-1), p53, BAX and loading control (GAPDH) protein levels in heart lysates from S, M and EM mice. Quantification of COL-1, p53 and BAX levels relative to GAPDH (*n* = 3). All data are presented as mean ± s.d. One-way ANOVA. ns, *P* > 0.05; *P < 0.05, **P < 0.01, ***P < 0.001, ****P < 0.0001.

Mice with EC-specific PRMT7 ablation subjected to MI (EndoKO-MI) exhibited a transient increase in HR and standard deviation (s.d.) of the interbeat interval of normal sinus beats (SDNN) at 1 week after MI, followed by a significant HR decline at 3 weeks after MI, suggesting worsened cardiac dysfunction (Fig. 2a and Supplementary Fig. 3b). Echocardiography revealed that EndoKO-MI mice had a more severe reduction in EF and FS compared with sham-operated (sham) mice (Fig. 2b, c). LV mass was notably reduced in EndoKO-MI mice, with increased LV chamber size and wall thinning, indicating greater LV remodeling (Fig. 2d and Supplementary Fig. 3c). Hemodynamic analysis showed decreased aortic acceleration time in EndoKO-MI mice, further supporting compromised cardiac function (Supplementary Fig. 3d). EndoKO-MI mice also displayed impaired right ventricular compensation, with reduced function compared with MI mice (Supplementary Fig. 3e). Increased Evans blue accumulation in EndoKO-MI hearts suggested greater vascular permeability and leakage (Fig. 2e). In addition, EndoKO-MI mice had elevated heart-to-body weight ratios and larger cardiomyocyte cross-sectional areas (Fig. 2f and Supplementary Fig. 4a).

Structural and molecular analysis revealed worsened cardiac injury in EndoKO-MI hearts. Connexin 43 staining showed widened gap junctions (Supplementary Fig. 4b,c), while H&E and Masson’s trichrome staining highlighted extensive necrosis and fibrosis (Fig. 2g, h). Apoptosis and cardiac damage markers (TUNEL-positive cardiomyocytes, p53 and BAX) were significantly increased in EndoKO-MI hearts (Fig. 2i, j), along with elevated collagen type 1 levels.

These findings suggest that endothelial PRMT7 deficiency exacerbates cardiac damage, increases vascular permeability and impairs cardiac recovery after MI.

### PRMT7 ablation in ECs altered the expression of genes associated with angiogenesis, cell proliferation and apoptosis after MI

To investigate the molecular changes underlying the aggravated cardiac dysfunction caused by PRMT7 ablation in ECs, RNA sequencing analysis was performed on hearts from sham, MI and EndoKO-MI mice 3 weeks after MI (Supplementary Fig. 5a). PCA revealed that the total gene expression profile of MI hearts exhibited considerable overlap with sham hearts, whereas EndoKO-MI hearts showed minimal overlap with sham hearts (Supplementary Fig. 5b). Further PCA analysis focused on specific pathway-related genes revealed that sham and MI displayed overlap in angiogenesis and cell death, whereas EndoKO-MI exhibited no overlap with sham in these pathways. Differential expression analysis identified 298 and 622 notably altered genes in MI and EndoKO-MI hearts, respectively, compared with sham controls (Supplementary Fig. 5c). A comparison between MI and EndoKO-MI hearts identified 318 significantly altered genes. Gene-specific analysis revealed a downregulation of genes associated with angiogenesis, cell cycle and cell differentiation alongside an upregulation of genes related to apoptosis in EndoKO-MI hearts compared with MI hearts (Supplementary Fig. 5d). Together, these findings suggest that PRMT7 ablation in ECs exacerbates MI-induced cardiomyopathy by dysregulating gene expression related to angiogenesis, cell proliferation and apoptotic processes.

### PRMT7 is expressed in ECs with angiogenic characteristics in MI hearts and is required for the proliferation and survival of ECs

**Fig. 3:**
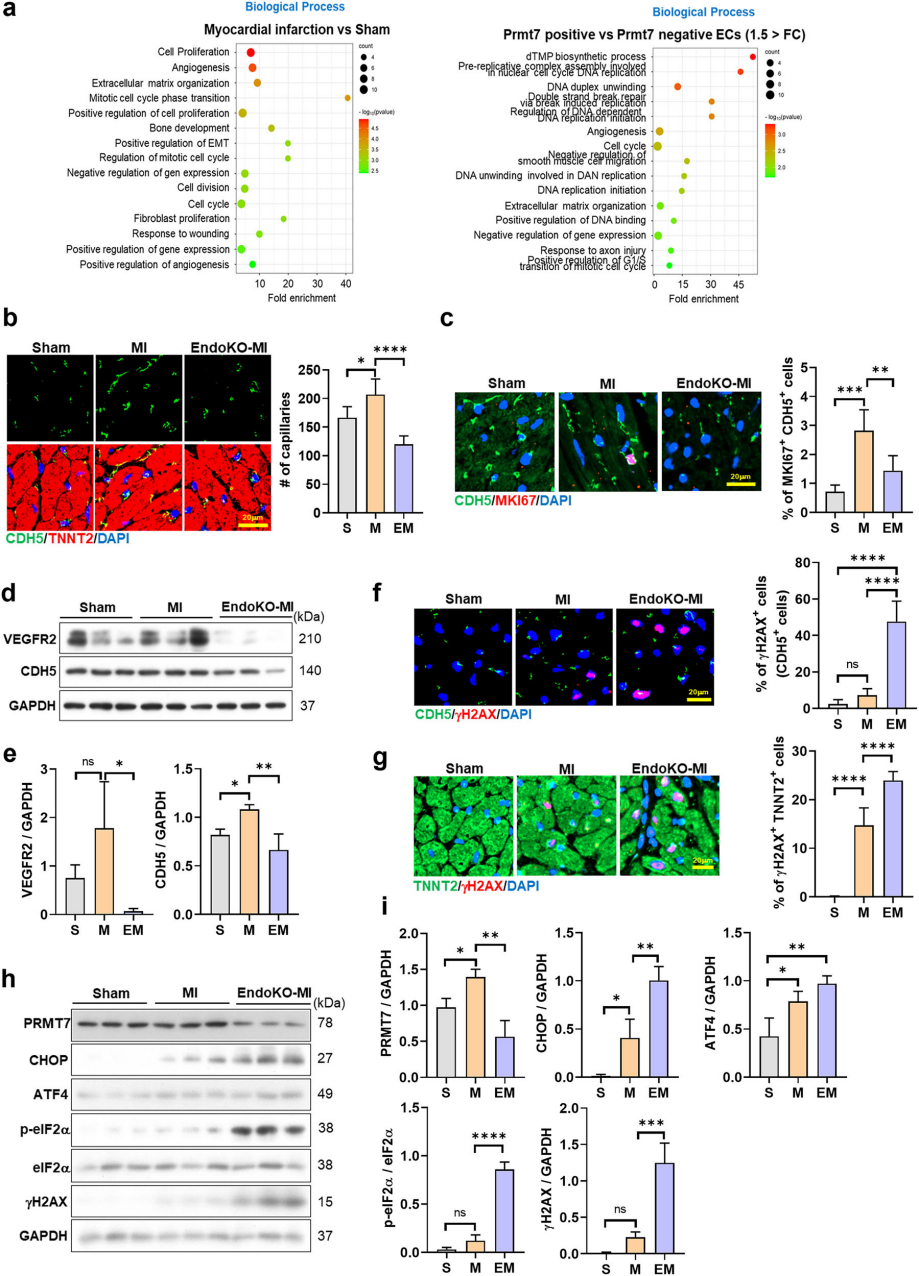
PRMT7 ablation in ECs alters angiogenesis, cell proliferation and apoptosis-related gene expression after MI. **a** Representative GO terms of enriched biological process on the upregulated genes from murine MI samples (GSE201947). **b** Representative images of heart sections stained for CDH5 (green) and TNNT2 (red) and counterstained with DAPI (blue). Scale bar, 20 µm. Quantification of capillary number in the heart tissues of sham (S), MI (M) or EndoKO-MI (EM) mice. **c** Representative images of heart sections stained for CDH5 (green) and MKI67 (red) and counterstained with DAPI (blue). Scale bar, 20 µm. Quantification of the percentage of MKI67^+^ CDH5^+^ cells in the heart tissues. **d** Immunoblot analysis of VEGFR2, CDH5 and GAPDH protein levels in heart lysates. **e** Quantification of VEGFR2 and CDH5 protein levels relative to GAPDH (*n* = 3). **f** Representative images of heart sections stained for CDH5 (green) and γH2AX (red) and counterstained with DAPI. Scale bar, 20 µm. Quantification of the percentage of γH2AX^+^CDH5^+^ cells in the heart tissues. **g** Representative images of heart sections stained for TNNT2 (green) and γH2AX (red) and counterstained with DAPI (blue). Scale bar, 20 µm. Quantification of the percentage of γH2AX^+^ cardiomyocytes in the heart tissues. **h** Immunoblot analysis of PRMT7, CHOP, ATF4, p-eIF2α, eIF2α, γH2AX and GAPDH protein levels in heart lysates. **i** Quantification of p-eIF2α relative to eIF2α, PRMT7, CHOP, ATF4 and γH2AX relative to GAPDH (*n* = 3). All data are presented as mean ± s.d. One-way ANOVA. ns, *P* > 0.05; *P < 0.05, **P < 0.01, ***P < 0.001, ****P < 0.0001.

Analysis of previously published scRNA-seq data from murine hearts 7 days after MI (GSE201947)^43^ revealed a strong upregulation of genes linked to cell proliferation and angiogenesis (Fig. 3a). At peak *Prmt7* expression in ECs, *Prmt7*-positive ECs showed enrichment in genes related to dTMP biosynthesis, DNA replication, angiogenesis, cell cycle regulation and extracellular matrix organization, supporting the role of PRMT7 in EC proliferation and angiogenesis after MI.

To further investigate this, we examined revascularization in EndoKO-MI hearts. Three weeks after MI, MI hearts exhibited a significant increase in CDH5-positive EC density compared with sham hearts, indicating enhanced revascularization (Fig. 3b). By contrast, EndoKO-MI hearts showed reduced CDH5-positive ECs, suggesting impaired revascularization. This decline correlated with lower EC proliferation, as indicated by reduced MKI67/CDH5 co-positivity in EndoKO-MI hearts (Fig. 3c).

Consistently, VEGFR2 and CDH5 expression levels were elevated in MI hearts but significantly reduced in EndoKO-MI hearts (Fig. 3d, e). DNA damage analysis revealed increased γH2AX-positive ECs in EndoKO-MI hearts, whereas MI hearts exhibited no significant EC-specific increase (Fig. 3f). Both MI and EndoKO-MI hearts had elevated γH2AX-positive cardiomyocytes, but the percentage was higher in EndoKO-MI hearts, suggesting greater cellular stress (Fig. 3g). These findings indicate that PRMT7 supports revascularization by enhancing EC proliferation and reducing stress-induced DNA damage after MI.

As ER stress contributes to endothelial dysfunction in cardiovascular diseases^46^, we analyzed its involvement. The public MI mouse model database 4, 7 and 14 days (GSE201947)^43^ after MI showed parallel upregulation of ER stress-related genes with increased *Prmt7* expression (Supplementary Fig. 6). Three weeks after MI, heart lysates from MI mice showed increased p-eIF2α, ATF4 and CHOP levels compared with sham hearts (Fig. 3h,i). Notably, EndoKO-MI hearts exhibited significantly higher levels of these ER stress markers, suggesting an exacerbated ER stress response.

These findings indicate that PRMT7 plays a crucial role in suppressing ER stress, promoting EC survival and enhancing angiogenesis in MI hearts, underscoring its importance in cardiac recovery after MI.

### Overexpression of PRMT7 alleviates TNF-α-induced ER stress and cell death in ECs

**Fig. 4:**
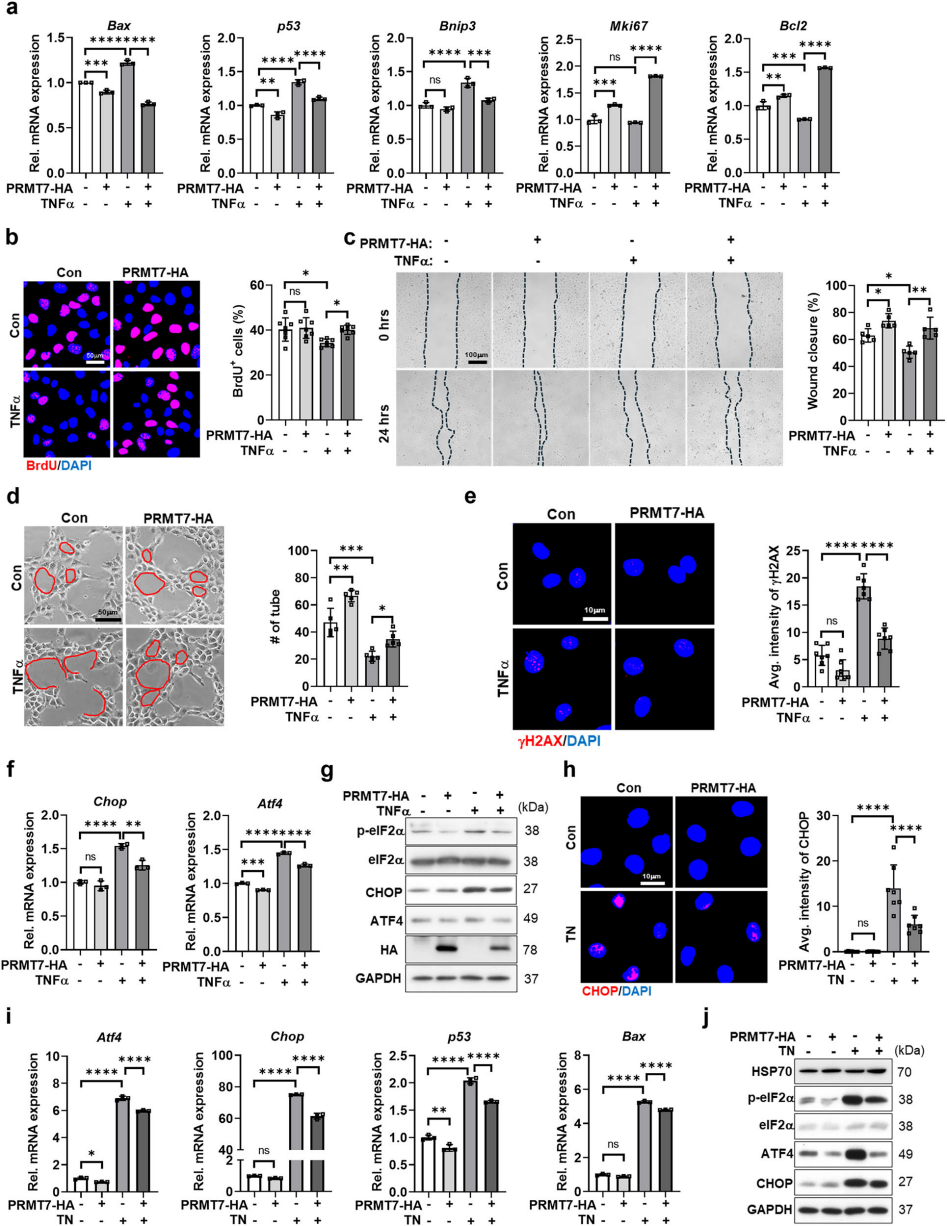
Overexpression of PRMT7 attenuates TNF-α-induced ER stress and cell death in C166 cells. **a**–**g** C166 cells were transfected with either pcDNA3.1 vector or pcDNA3.1 carrying *Prmt7*-HA to induce PRMT7 overexpression (PRMT7-HA) for 8 h. After transfection, C166 cells were treated with TNF-α (50 ng/ml) for 24 h: **a** qRT-PCR analysis of *Bax*, *p53*, *Bnip3*, *Mki67* and *Bcl*2 mRNA expression in C166 cells. **b** Representative images of BrdU immunostaining (red) and counterstained with DAPI (blue) in C166 cells. Scale bar, 50 µm. Quantification of the percentage of BrdU^+^ cells. **c** Representative images of the scratch wound healing assay in transfected C166 cells before treatment with TNF-α (0 h) and after treatment with TNF-α for 24 h. Scale bar, 100 µm. Quantification of percentage of wound closure from the scratch wound healing assay. **d** Representative images of the tube formation assay in C166 cells. Scale bar, 50 µm. Quantification of the tube number. **e** Representative images of C166 cells stained for DNA damage (γH2AX, red) and counterstained with DAPI (blue). Scale bar, 10 µm. Quantification of the average intensity of γH2AX in C166 cells. **f** qRT-PCR analysis of *Chop* and *Atf4* mRNA expression in C166 cells. **g** Immunoblot analysis of p-eIF2α, eIF2α, CHOP, ATF4, HA (tag protein) and GAPDH protein levels in C166 cells. **h–j** C166 cells were transfected with either pcDNA3.1 vector or pcDNA3.1 carrying *Prmt7*-HA to induce PRMT7 overexpression (PRMT7-HA) for 8 h, followed by treatment with TN (10 µg/ml) for 24 h: **h** Representative images of C166 cells stained for CHOP (red) and counterstained with DAPI (blue). Scale bar, 10 µm. Quantification of the average intensity of CHOP. **i** qRT-PCR analysis of *Atf4*, *Chop*, *p53* and *Bax* mRNA expression in C166 cells. **j** Immunoblot analysis of HSP70, p-eIF2α, eIF2α, ATF4, CHOP and GAPDH protein levels in C166 cells. All data are presented as mean ± s.d. One-way ANOVA. ns, *P* > 0.05; *P < 0.05, **P < 0.01, ***P < 0.001, ****P < 0.0001.

To confirm the protective role of PRMT7 in ECs under stress conditions, ECs were transfected with either control or PRMT7-overexpressing vectors, followed by TNF-α treatment. PRMT7 overexpression attenuated the expression of cell death markers (*Bax*, *Bnip3* and *p53*) while increasing the expression of the survival and proliferation markers *Bcl2* and *Mki67* in response to TNF-α compared with controls (Fig. 4a). Consistently, PRMT7 overexpression restored BrdU incorporation and improved the wound healing capacity of ECs affected by TNF-α treatment (Fig. 4b, c). Similarly, PRMT7 overexpression mitigated the impaired tube formation triggered by TNF-α (Fig. 4d). Moreover, PRMT7 overexpression reduced γH2AX accumulation and downregulated ER stress-related signaling pathways induced by TNF-α treatment (Fig. 4e–g). A similar protective effect was observed under TN treatment, where PRMT7 overexpression attenuated CHOP accumulation and suppressed the expression of ER stress and cell death regulators (Fig. 4h–j). In addition, HSP70 protein levels were elevated in cells expressing PRMT7, further supporting a positive regulatory role of PRMT7 in modulating HSP70 expression under cellular stress conditions. These findings suggest that PRMT7 overexpression may serve as an effective strategy to prevent EC dysfunction and enhance revascularization after MI.

### Bindarit enhances PRMT7 expression and attenuates TNF-α-induced EC dysfunction

**Fig. 5:**
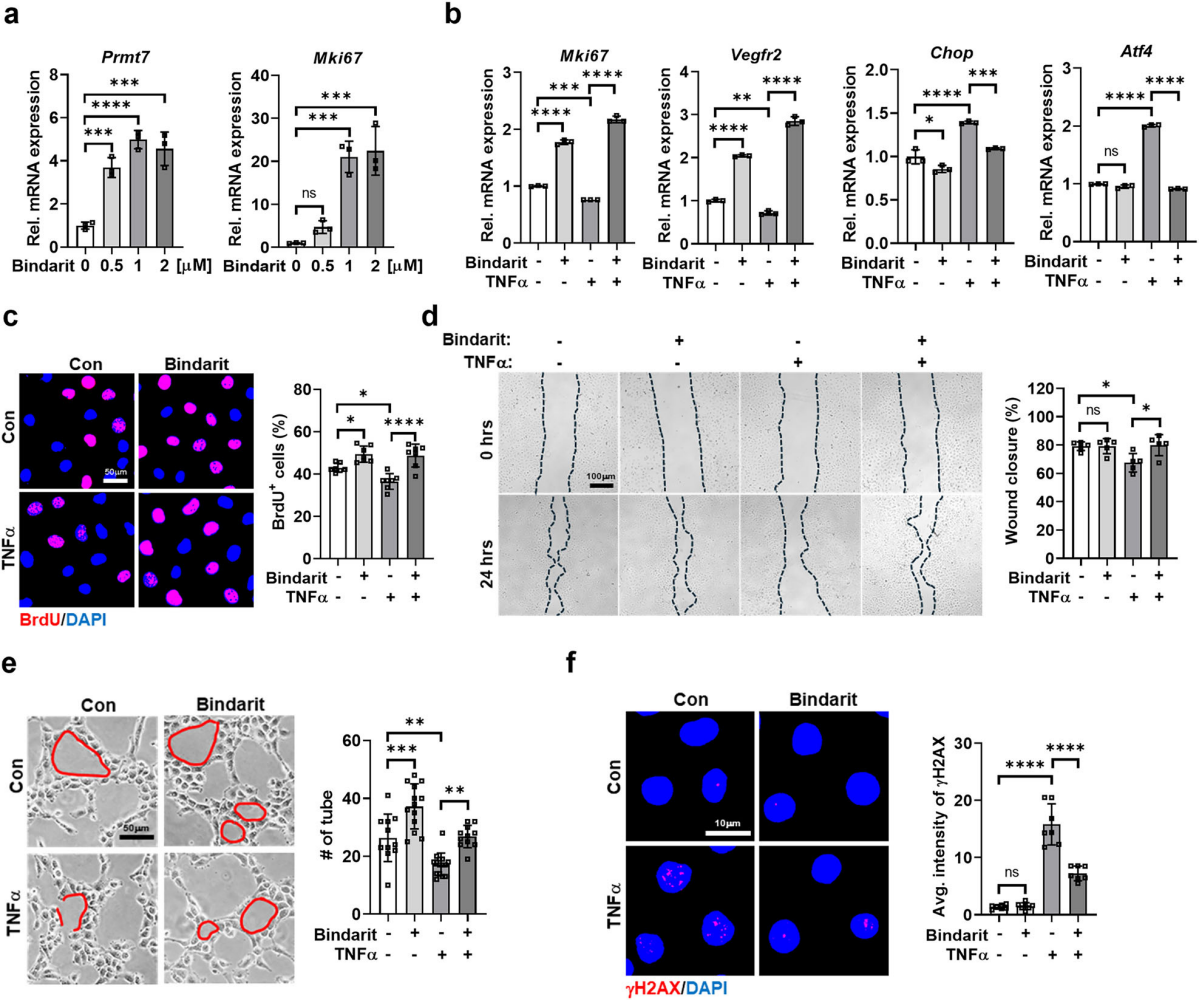
Bindarit treatment mitigates TNF-α-induced endothelial dysfunction in C166 cells. **a** qRT-PCR analysis of *Prmt7* and *Mki67* mRNA expression in C166 cells treated with DMSO or bindarit at concentrations of 0.5, 1 or 2 µM. **b**–**f** C166 cells were pretreated with bindarit (500 nM) for 1 h and subsequently treated with TNF-α (50 ng/ml) for 24 h: **b** qRT-PCR analysis of *Mki67*, *Vegfr2*, *Chop* and *Atf4* mRNA expression in C166 cells treated with DMSO, bindarit (500 nM), TNF-α (50 ng/ml) or a combination of bindarit and TNF-α for 24 h. **c** Representative images of C166 cells stained for BrdU (red) and counterstained with DAPI (blue). Scale bar, 50 µm. Quantification of the percentage of BrdU-positive cells. **d** Representative images of the scratch wound healing assay in bindarit-pretreated C166 cells before treatment with TNF-α (0 h) and after treatment with TNF-α for 24 h. Scale bar, 100 µm. Quantification of the wound closure percentage from the scratch wound healing assay. **e** Representative images of the tube formation assay in C166 cells. Scale bar, 50 µm. Quantification of the tube number. **f** Representative images of C166 cells stained for DNA damage (γH2AX, red) and counterstained with DAPI (blue). Scale bar, 10 µm. Quantification of the average intensity of γH2AX in C166 cells. All data are presented as mean ± s.d. One-way ANOVA. ns, *P* > 0.05; *P < 0.05, **P < 0.01, ***P < 0.001, ****P < 0.0001.

We next investigated the protective effects of bindarit on EC stress in response to TNF-α. Bindarit was identified as an inducer of PRMT7 expression using a luciferase reporter controlled by the *Prmt7* promoter (data not shown). Consistent with screening data, bindarit treatment increased *Prmt7* and *Mki67* expression in ECs, with maximal induction at 1 µM (Fig. 5a). While bindarit is known to inhibit the pro-inflammatory factor CCL-2, this effect occurs only at much higher concentrations (300 µM)^47^. At the 500 nM concentration used in our study, bindarit did not affect *Ccl-2* and *Icam-1* expression under basal or TNF-α-induced conditions (Supplementary Fig. 7a). A previous study has shown that bindarit enhances p38 MAPK activation in the cytosol^48^, which in turn induces ATF3, a transcription factor involved in cellular stress responses^49^. In our study, bindarit treatment increased phosphorylated p38 MAPK and *Atf3* expression, whereas pharmacological inhibition of p38 MAPK with SB203580 abrogated bindarit-induced upregulation of *Prmt7* and *Atf3* (Supplementary Fig. 7b–d). These findings suggest that low-dose bindarit may promote *Prmt7* expression via p38 MAPK signaling activation.

Similar to PRMT7 overexpression, bindarit significantly increased angiogenic factors such as *Mki67* and *Vegfr2*, even under TNF-α-induced stress (Fig. 5b). In addition, it suppressed TNF-α-induced upregulation of ER stress-related genes *Chop* and *Atf4*. Consistent with increased *Mki67*, bindarit enhanced the number of BrdU-positive ECs and counteracted the TNF-α-induced decline in proliferation (Fig. 5c).

Functionally, bindarit improved EC migration and angiogenesis, and mitigated TNF-α-induced impairments in wound healing and tube formation (Fig. 5d, e). Furthermore, bindarit reduced TNF-α-induced γH2AX accumulation, indicating decreased DNA damage (Fig. 5f).

These findings suggest that bindarit protects ECs from inflammatory stress by promoting proliferation, angiogenesis and DNA repair, making it a promising therapeutic candidate for preventing EC dysfunction in cardiovascular diseases, including MI.

### Bindarit improves cardiac recovery after MI

**Fig. 6:**
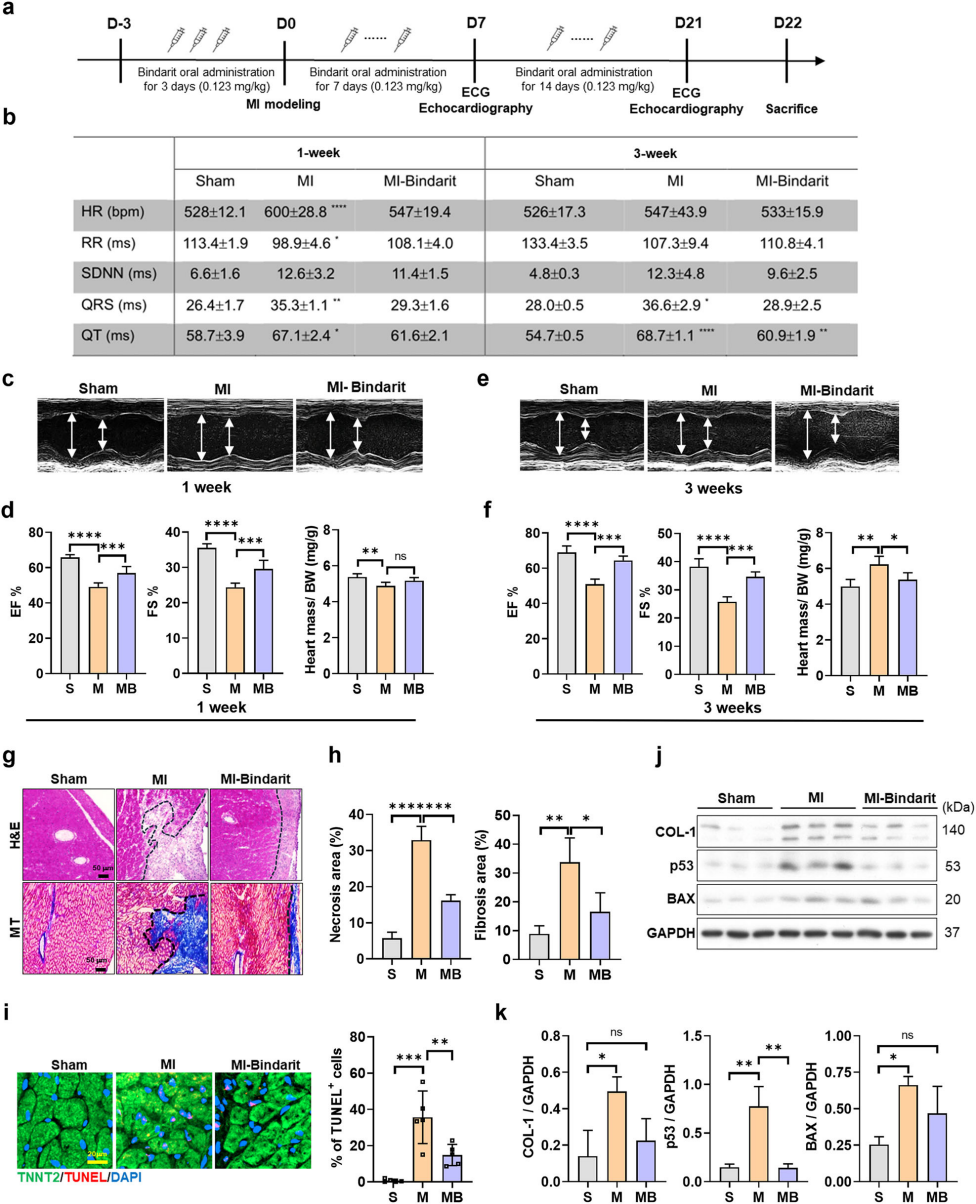
Bindarit improves cardiac recovery in MI mice. **a** The experimental scheme for the oral administration of bindarit in MI model. **b** Electrocardiography parameter analysis 1 week and 3 weeks after MI. **c** Representative images of echocardiography of mice 1 week after MI. **d** Analysis of electrocardiography parameters and relative heart mass to body weight of sham (S), MI (M) and MI-bindarit (MB) 1 week after MI. **e**, Representative images of echocardiography of mice 3 weeks after MI. **f** Analysis of electrocardiography parameters and relative heart mass to body weight 3 weeks after MI. **g** H&E staining and Masson’s trichrome (MT) staining of hearts 3 weeks after MI. Scale bar, 50 µm. **h** Quantification of the percentage of necrosis and fibrosis area in heart tissues. **i** Representative images of heart sections stained for TUNEL (red) and TNNT2 (green) and counterstained with DAPI (blue). Scale bar, 20 µm. Quantification of the percentage of TUNEL-positive cells. **j** Immunoblot analysis of collagen type 1 (COL-1), p53, BAX and GAPDH protein levels in heart lysates. **k** Quantification of COL-1, p53, BAX relative to GAPDH (*n* = 3). All data are presented as mean ± s.d. One-way ANOVA. ns, *P* > 0.05; *P < 0.05, **P < 0.01, ***P < 0.001, ****P < 0.0001.

We next evaluated the effects of bindarit on cardiac recovery after MI (Fig. 6a). Electrocardiography 1 and 3 weeks after MI showed improved cardiac function in bindarit-treated mice, as indicated by better HR and cardiac rhythm parameters (RR interval, s.d. of normal-to-normal intervals, QRS and QT durations) compared with vehicle-treated MI mice (Fig. 6b).

One week after MI, echocardiography revealed a significant reduction in EF and FS in both MI groups compared with sham controls. However, the decline was less severe in bindarit-treated mice, and they exhibited preserved relative heart mass after MI (Fig. 6c, d).

By 3 weeks post MI, bindarit-treated mice showed near-normal cardiac function, whereas vehicle-treated MI mice continued to display reduced EF and FS (Fig. 6e, f). In addition, the increased relative cardiac mass observed in vehicle-treated MI mice was mitigated by bindarit.

Histological analysis 3 weeks after MI demonstrated that bindarit reduced cardiomyocyte hypertrophy, as indicated by smaller cross-sectional areas, and alleviated necrotic and fibrotic tissue in MI hearts (Fig. 6g, h and Supplementary Fig. 8a). TUNEL staining showed a significant reduction in apoptotic cardiomyocytes in bindarit-treated hearts compared with vehicle-treated MI hearts (Fig. 6i). This was further supported by decreased expression of apoptotic markers p53 and BAX in bindarit-treated hearts, whereas vehicle-treated hearts exhibited elevated levels (Fig. 6j, k). In addition, bindarit treatment reduced fibrosis markers (collagen type 1) and heart failure markers (*Nppa* and *Nppb*) compared with vehicle-treated MI hearts (Supplementary Fig. 8b). Inflammation and fibrosis levels were examined in liver tissue to evaluate systemic effects of MI (Supplementary Fig. 8c). Although bindarit treatment attenuated *Mmp12* expression in MI livers, levels of other inflammatory and fibrotic markers showed no significant changes in both MI and bindarit-treated MI compared with sham.

These findings suggest that bindarit protects against cardiac damage, reduces apoptosis and promotes recovery after MI.

### Bindarit treatment improves revascularization by promoting angiogenic signaling and suppressing ER stress signaling

**Fig. 7:**
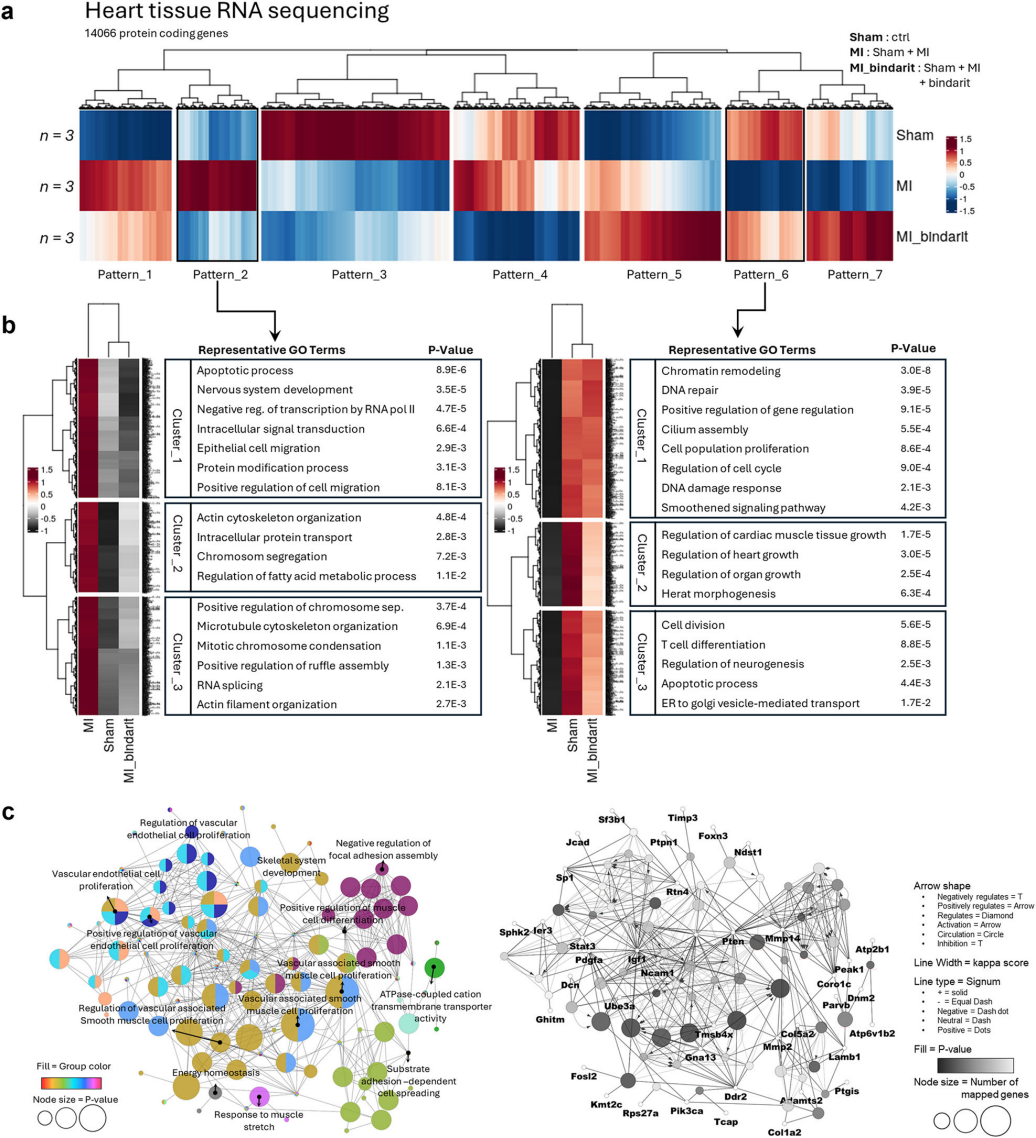
Bindarit restores gene expression patterns in MI mice. **a** Clustered heatmap analysis of differential expressions of protein-coding genes in sham, MI and MI-bindarit (*n* = 3). **b** Representative GO terms for pattern 2 (left) and pattern 6 (right) from **a**. **c** Upregulated genes (with a normalized data value of 6.0 or higher) were imported into Cytoscape for GO clustering using ClueGO plugin. GO terms are represented as nodes, and the node size represents the term enrichment significance. Groupings with fewer than two connections were excluded from the final list of networks. Representative genes are shown for upregulated subclusters in MI-bindarit hearts.

To investigate the molecular mechanisms underlying the cardioprotective effects of bindarit, we performed total RNA sequencing on heart tissues from three groups, sham, vehicle-treated MI and bindarit-treated MI (MI-bindarit). Transcriptome analysis of 14,066 protein-coding genes revealed seven distinct patterns of differential gene expression (Fig. 7a). Notably, gene expression profiles in MI-bindarit hearts resembled those of sham in patterns 2 and 6, with down- and upregulated genes, respectively (Fig. 7b). By contrast, MI hearts displayed opposite trends compared with sham in these patterns. GO analysis of pattern 2 revealed a downregulation of genes associated with apoptotic process and actin cytoskeleton organization, while pattern 6 was enriched in pathways related to DNA repair, cell population proliferation, cell cycle, heart growth and cell division. Further clustering analysis using Cytoscape demonstrated that upregulated genes in MI-bindarit hearts were involved in endothelial and smooth muscle cell proliferation, energy homeostasis, muscle stretch response, ATPase-coupled transport and focal adhesion regulation (Fig. 7c).

**Fig. 8:**
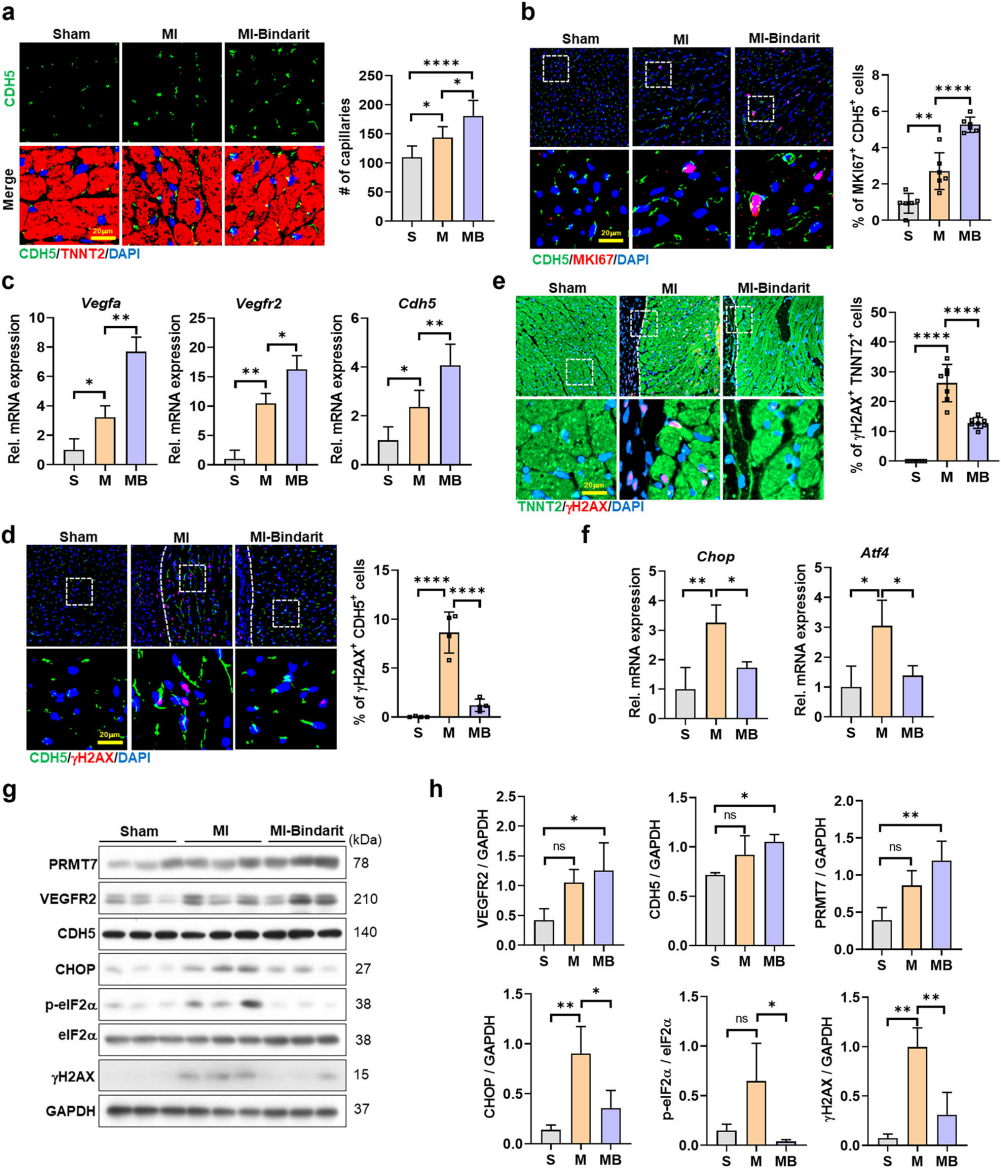
Bindarit administration promotes revascularization by promoting angiogenic signaling and suppressing ER stress signaling in MI mice. **a** Representative images of heart sections stained for CDH5 (green) and TNNT2 (red) and counterstained with DAPI (blue). Scale bar, 20 µm. Quantification of capillary numbers in the heart of sham (S), MI (M) or MI-bindarit (MB) mice. **b** Representative images of heart sections stained for CDH5 (green) and MKI67 (red) and counterstained with DAPI (blue). Scale bar, 20 µm. Quantification of the percentage of MKI67-positive ECs in the heart tissues. **c** qRT-PCR analysis of *Vegfa*, *Vegfr2* and *Cdh5* mRNA expression of the heart tissue (*n* = 3). **d** Representative images of heart sections stained for CDH5 (green) and γH2AX (red) and counterstained with DAPI (blue). Scale bar, 20 µm. Quantification of the percentage of γH2AX^+^CDH5^+^ cells in the heart tissues. **e** Representative images of heart sections stained for TNNT2 (green) and γH2AX (red) and counterstained with DAPI (blue). Scale bar, 20 µm. Quantification of the percentage of γH2AX^+^TNNT2^+^ cells in the heart tissues. **f** qRT-PCR analysis of *Chop* and *Atf4* mRNA expression of heart tissues (*n* = 3). **g** Immunoblot analysis of PRMT7, VEGFR2, CDH5, CHOP, p-eIf2α, eIF2α, γH2AX and GAPDH protein levels in heart lysates. **h**, Quantification of PRMT7, VEGFR2, CDH5, CHOP and γH2AX relative to GAPDH, and p-eIF2α relative to eIF2α (*n* = 3). All data are presented as mean ± s.d. One-way ANOVA. ns, *P* > 0.05; *P < 0.05, **P < 0.01, ***P < 0.001, ****P < 0.0001.

Next, we validated these transcriptomic changes through experimental analyses, confirming their functional significance. Immunostaining showed increased EC density in infarcted hearts, with MI-bindarit hearts exhibiting a further elevation compared with vehicle-treated MI hearts (Fig. 8a). This increase in revascularization correlated with a higher number of MKI67-positive proliferating ECs (Fig. 8b), aligning with transcriptome findings indicating enhanced endothelial proliferation. Furthermore, both mRNA and protein levels of key angiogenic markers *Vegfa*, *Vegfr2* and *Cdh5* were elevated in MI hearts and further upregulated by bindarit treatment (Fig. 8c), supporting improved revascularization and cardiac recovery.

Consistent with the transcriptomic identification of DNA repair pathways in pattern 6, bindarit reduced γH2AX accumulation, a marker of DNA damage, in ECs and cardiomyocytes in MI hearts (Fig. 8d, e), suggesting protection against MI-induced cellular stress. In addition, bindarit attenuated the MI-induced increase in *Atf4* and *Chop* transcripts (Fig. 8f). Protein analysis confirmed that bindarit treatment increased PRMT7, VEGFR2 and CDH5 levels while significantly reducing p-eIF2α, CHOP and γH2AX levels compared with vehicle-treated MI hearts (Fig. 8g, h).

These findings collectively suggest that bindarit promotes cardiac recovery after MI by inducing PRMT7 expression, enhancing angiogenic responses and suppressing ER stress-related pathways (Supplementary Fig. 9).

In summary, endothelial PRMT7 deficiency worsens MI-induced cardiac damage due to dysregulated ER stress and increased cell death. Conversely, PRMT7 induction via bindarit treatment enhances cell survival and revascularization, highlighting PRMT7 as a promising therapeutic target for mitigating ischemia-induced cardiomyopathy.

## Discussion

This study highlights PRMT7 as a critical regulator of EC function during inflammatory stress and MI-induced cardiac repair. We show that PRMT7 expression is upregulated in ECs in response to TNF-α or TN, and its inhibition compromises EC viability, angiogenesis and tube formation. These findings establish PRMT7 as a key protector of ECs under stress. Prior studies link PRMT7 to unfolded protein response regulation through GRP78/BiP and eIF2α methylation^34,35^. Our data suggest that PRMT7 mitigates ER stress to prevent apoptosis during prolonged cellular stress.

Among potential PRMT7 targets involved in ER stress responses, p38 MAPK and HSP70 are of particular interest. Our previous works demonstrated that PRMT7 methylates p38 MAPK in muscle cells to regulate myogenic differentiation^33^. Given the established role of p38 MAPK in ER stress^50^, it is plausible that PRMT7 modulates ER stress responses in ECs through p38 MAPK activation. Moreover, prior studies have shown that PRMT7 directly methylates HSP70 at arginine 469 residue, thereby regulating its activity in cellular stress responses^34^. In this study, inhibition of PRMT7 with SGC8158 reduced HSP70 protein levels, whereas PRMT7 overexpression restored HSP70 expression under TN-induced stress. While these findings suggest a positive regulatory role for PRMT7 in modulating HSP70 expression during stress responses, further investigation is needed to determine whether PRMT7 directly methylates and regulates HSP70 in ECs in response to cellular stress, including ER stress.

Interestingly, VEGFA, which transiently induces ER stress during proliferation, did not alter PRMT7 expression, whereas TNF-α—an inflammatory cytokine—induced its expression. This suggests that PRMT7 functions as a protective response to inflammation during MI. Consistently, PRMT7-deficient (EndoKO) mice exhibited worsened cardiac dysfunction after MI, with increased expression of ER stress and apoptotic markers, including p-eIF2α, ATF4, CHOP, p53 and BAX. These findings suggest that PRMT7 is essential for maintaining ER homeostasis in ECs and preventing maladaptive UPR-induced apoptosis.

Effective cardiac repair requires early revascularization to restore blood flow and limit damage^51,52^. During the early stages of MI, ECs are exposed to various stress stimuli, including inflammation, oxidative stress and ER stress^53^. Under these conditions, ECs that successfully adapt and survive exhibit upregulation of pro-angiogenic markers, such as VEGFR2, thereby promoting vessel sprouting and angiogenesis essential for cardiac repair and tissue regeneration^54^. In our study, we demonstrate that PRMT7-deficient ECs exhibit impaired angiogenesis, characterized by reduced vessel density, downregulation of angiogenic markers, including VEGFR2, and increased apoptosis. This impairment leads to insufficient revascularization after MI. Conversely, PRMT7 overexpression or pharmacological induction with bindarit enhanced EC proliferation, migration and tube formation, promoting revascularization and tissue repair. The impaired angiogenic response in EndoKO-MI hearts further underscores the essential role of PRMT7 in EC-driven revascularization. While these findings establish a functional link between PRMT7 and angiogenesis, further investigation is required to elucidate the direct mechanisms by which PRMT7 regulates angiogenic signaling under ischemic conditions.

Beyond angiogenesis, PRMT7 appears to modulate inflammation. While TNF-α drives an essential inflammatory response for tissue repair, excessive inflammation exacerbates damage^55,56^. Bindarit, a small molecule identified in this study as a PRMT7 inducer, selectively suppressed lipopolysaccharide-induced IL-6, IL-1β and CCL-2 expression without affecting basal cytokine levels^57^. Unlike its known NF-κB inhibitory effects at high doses (≥300 μM)^58^, we observed significant PRMT7-inducing and ER stress-attenuating effects at much lower concentrations (0.5–1 μM). This suggests that bindarit modulates ER stress through PRMT7 induction while preserving homeostasis.

Bindarit has been reported to enhance the activation of cytosolic p38 MAPK^48^, a signaling pathway known to induce ATF3 expression, a key transcription factor involved in cellular stress responses^49^. Our findings suggest that low-dose bindarit predominantly acts via p38 MAPK activation, rather than through NF-κB inhibition. In our results, bindarit treatment increased levels of phosphorylated p38 MAPK, supporting its role in p38 MAPK activation. Furthermore, inhibition of p38 MAPK with SB203580 blocked bindarit-induced *Prmt7* expression. Notably, treatment with bindarit at 0.5 μM did not alter the expression of inflammatory markers. Taken together, these findings support the hypothesis that bindarit upregulates *Prmt7* through p38 MAPK signaling. However, further studies are needed to determine whether bindarit directly interacts with p38 MAPK and to validate p38 MAPK-dependent ATF3 binding at the *Prmt7* promoter.

PRMT7 may have broader cardioprotective roles beyond ECs. Prior studies show that PRMT7 deletion in cardiomyocytes induces hypertrophy and fibrosis via dysregulated β-catenin signaling^31^. In addition, PRMT7 overexpression attenuates angiotensin II-induced cardiac hypertrophy, whereas PRMT7 deletion exacerbates it. Combined with our findings, these data suggest that PRMT7 protects both ECs and cardiomyocytes by regulating ER stress, apoptosis and fibrosis. The observation that *PRMT7* peaks in the ischemic zone during the acute MI phase and normalizes during fibrosis further supports its time-sensitive role in early cardiac repair.

Our findings establish PRMT7 as a promising therapeutic target for modulating ER stress and promoting angiogenesis in ischemic cardiomyopathy. The identification of bindarit as a PRMT7 inducer provides a potential therapeutic approach. Notably, bindarit improved cardiac function, reduced fibrosis and increased capillary density after MI, effects associated with reduced ER stress markers (ATF4, CHOP and p-eIF2α) and DNA damage (γH2AX). These results suggest that bindarit enhances cardiac repair by reducing ER stress and directly promoting EC survival and function via PRMT7 induction.

Future studies should explore combining PRMT7-inducing agents like bindarit with standard antifibrotic and immunomodulatory therapies. Notably, a recent study reported a role for PRMT7 in promoting monocyte migration and inflammation in the context of chronic obstructive pulmonary disease^30^. By contrast, our findings reveal a protective role of PRMT7 in ECs under cellular stress, supporting its pro-survival and pro-angiogenic roles. These divergent observations underscore the tissue-specific complexity of PRMT7 activation in different disease contexts. Therefore, the therapeutic application of bindarit or other PRMT7-targeting strategies in MI should be approached cautiously, especially in patients with comorbidities such as chronic obstructive pulmonary disease, where PRMT7 activation may have pro-inflammatory consequences. Moreover, further studies are necessary to evaluate the systemic safety and efficacy of bindarit, including its impact on noncardiac tissues such as the lung and liver. Furthermore, the role of PRMT7 in other cardiovascular conditions, including heart failure with preserved EF and nonischemic cardiomyopathies, warrants further investigation.

In this study, MI was induced using the LAD coronary artery ligation model^37^, which primarily recapitulates ischemic injury-driven heart failure. Although this model is well established for studying ischemic heart disease, it has limitations in specifically evaluating endothelial dysfunction as a primary contributor to MI. A recent study introduced an alternative MI model that induces cardiac dysfunction through nonligation methods, selectively targeting endothelial dysfunction while minimizing ischemia-induced cardiac injury^59^. Incorporating this alternative MI model in future studies will be essential to more directly validate the role of endothelial PRMT7 in MI pathology.

In summary, we identify PRMT7 as a central regulator of EC stress response and angiogenesis in MI. Bindarit enhances cardiac recovery by promoting EC survival, revascularization and ER stress suppression. Targeting PRMT7 represents a promising therapeutic strategy for reducing ischemic injury and improving cardiac repair.

## Supplementary information

Supplementary Information

## Data Availability

All data associated with this study are present in the Article or its Supplementary Information. The datasets used and/or analyzed during the current study are available from the corresponding author on reasonable request.
