# Supplementary material for: Endothelial PRMT7 prevents dysfunction, promotes revascularization and enhances cardiac recovery post-myocardial infarction
Source: Exp Mol Med. 2025 Aug 5;57(8):1759–74. doi: 10.1038/s12276-025-01517-x (PMC12411622; doi:10.1038/s12276-025-01517-x)
Supplement: Supplementary file 1 — Supplementary Information [file 12276_2025_1517_MOESM1_ESM.pdf]

Supplementary Materials for

**Endothelial PRMT7 prevents dysfunction, promotes revascularization, and enhances cardiac recovery post-myocardial infarction**

Thi Thuy Vy Tran, Yan Zhang, Shibo Wei, Jinwoo Lee, Yideul Jeong, Tuan Anh Vuong, Sang-Jin Lee, Dongryeol Ryu, Gyu-Un Bae^*^, Jong-Sun Kang^*^

**This file includes:**

Supplementary Figures 1 to 9

Supplementary Tables 1 to 2

**Supplementary Figures**

**
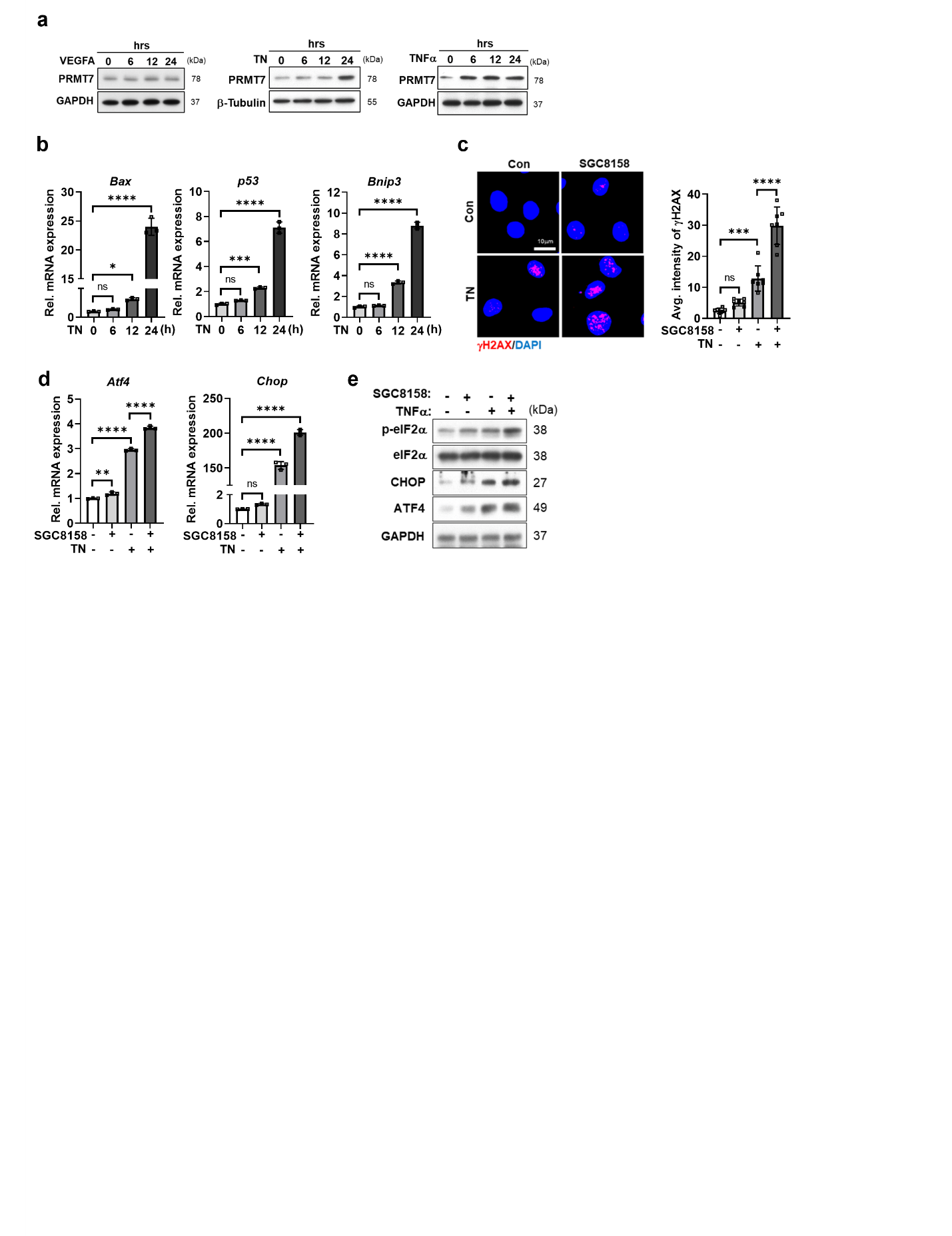
**

**Supplementary Fig. 1. a** Immunoblot analysis of PRMT7 and loading control (GAPDH or β- Tubulin) protein level of C166 cells treated with VEGFA (100ng/mL), Tunicamycin (TN, 10µg/mL), and TNF-α (50ng/mL) for different time durations (0, 6, 12, 24 hours). **b** Quantitative RT-PCR analysis of *Bax*, *p53*, and *Bnip3* mRNA expression of C166 cells treated with TN (10µg/mL) for different time durations (0, 6, 12, 24 hours). **c** Representative images of immunostaining for DNA damage (γH2AX, red), and counterstained with DAPI (blue) in C166 cells treated with DMSO, SGC8158 (1µM), TN (10µg/mL), or a combination of SGC8158 and TN for 24 hours. Scale bar, 10 µm. Quantification of average intensity of γH2AX in C166 cells. **d** Quantitative RT-PCR analysis of *Atf4* and *Chop* mRNA expression of C166 cells. **e** Immunoblot analysis of p-eIF2α, eIF2α, ATF4, CHOP, and GAPDH of C166 cells treated with DMSO, SGC8158 (1µM), TNF-α (50ng/mL), or a combination of SGC8158 and TNF-α for 24 hours. All data are presented as mean ± s.d. One-way ANOVA. ns for *P* > 0.05, **P* < 0.05, ***P* < 0.01, ****P* < 0.001, *****P* < 0.0001.


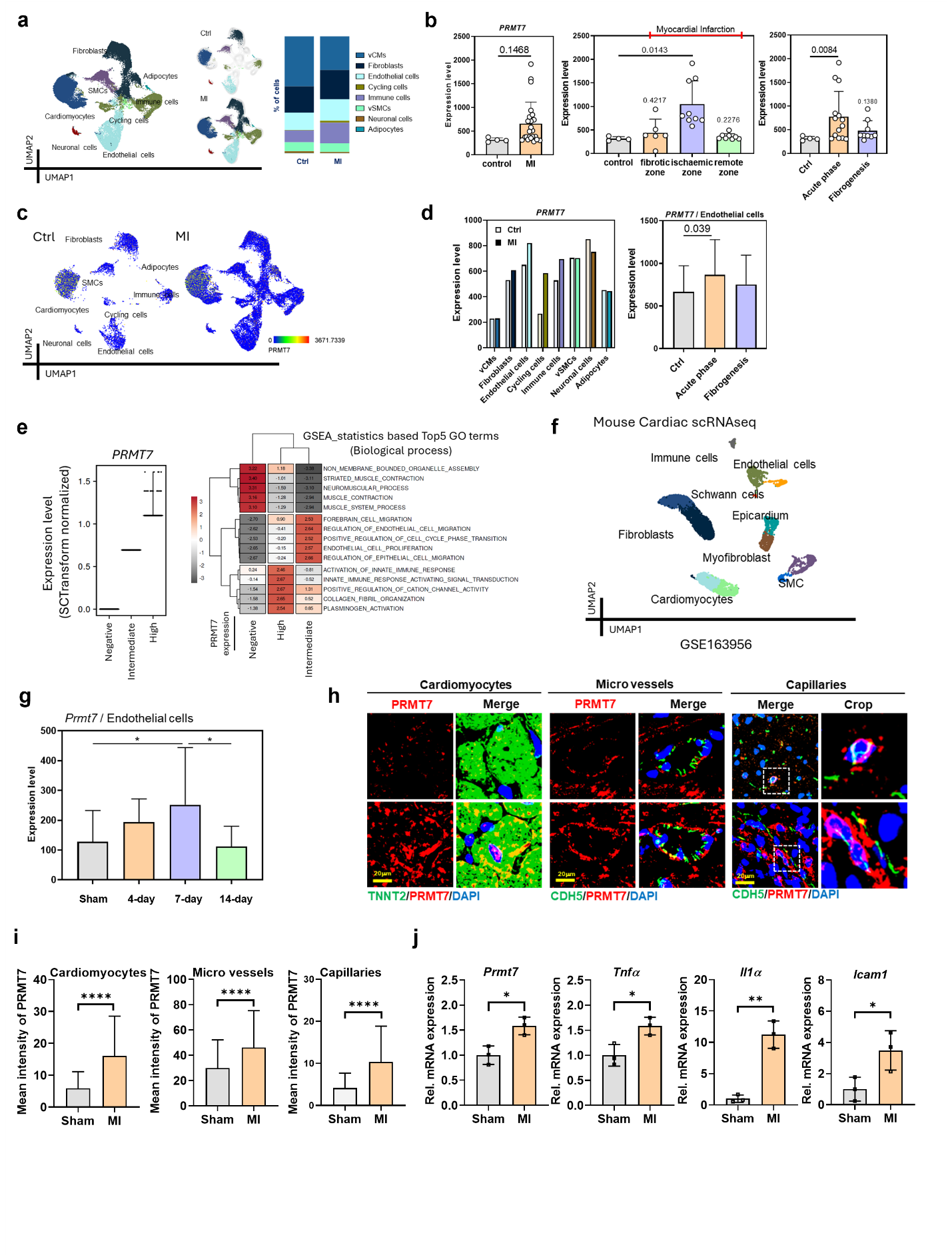


**Supplementary Fig. 2. a** Single cell transcriptomic analysis of human myocardial infarction (MI) database. Cell types were classified based on unique gene expression profiles following Seurat based uniform manifold approximation and projection (UMAP) clustering. Bar graph showing the proportion of *PRMT7* expressing cells. **b** Bar plot showing *PRMT7* expression levels in healthy individuals (control) and MI patients (left), across specific MI regions including fibrotic zone, ischemic zone, and remote zone (middle), and during different phases of MI: acute phase and fibrogenesis phase (right). **c** UMAP plots of *PRMT7* expressions in control (Ctrl) and MI samples. **d** Bar graph of *PRMT7* expression level across multiple cell types in control and MI (left), *PRMT7* expression in endothelial cells (ECs) during acute phase and fibrogenesis (right). **e** *PRMT7* expression level in ECs of human MI database (left), gene set enrichment analysis for negative-, intermediate-, and high-*PRMT7* expressing ECs (right). **f** Single cell transcriptomic analysis of murine MI samples from the GEO database (GSE201947). Cell types were classified based on their unique gene expression profiles using Seurat-based UMAP clustering. **g** Bar plot showing the expression levels of *Prmt7* in ECs from Sham and MI mice at day 4, day 7 and day 14 time points. **h** Representative images of cardiac sections stained for cardiomyocytes (TNNT2, green), ECs (CDH5, green), PRMT7 (red), and counterstained with DAPI (blue) in Sham and MI mouse model. Scale bar, 20 µm. **i** Quantification of PRMT7 intensity in cardiomyocytes, micro vessels, and capillaries. **j** Quantitative RT-PCR analysis of *Prmt7*, *Tnf-α*, *Il-1α*, and *Icam-1* mRNA expressions of heart samples from Sham and MI mice (n=3). All data are presented as mean ± s.d. Student t’test. * *P* < 0.05, ** *P* < 0.01, **** *P* <0.0001.
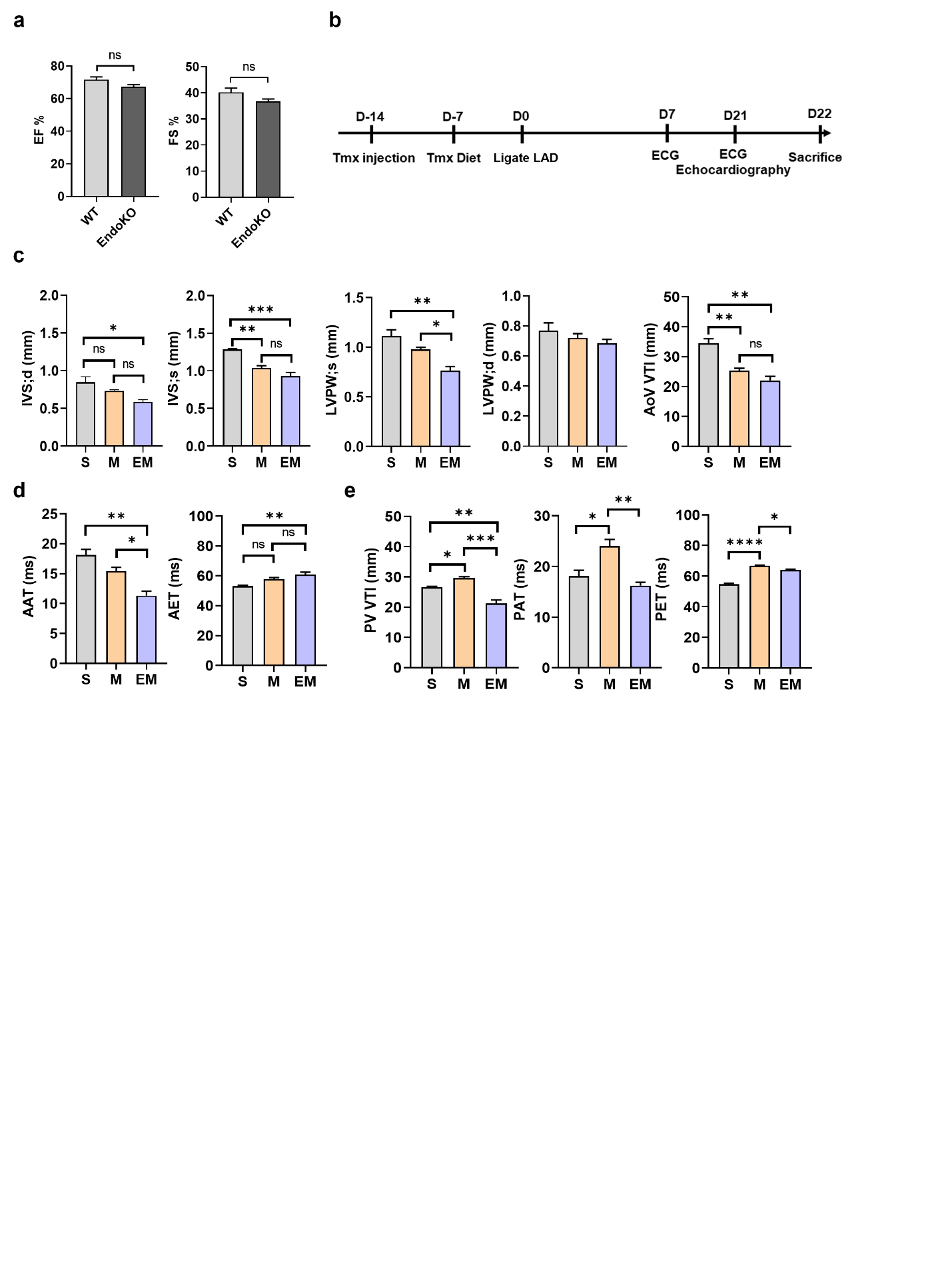


**Supplementary Fig. 3. a** Echocardiographic parameters of wild type (WT) and endothelial cell (EC)-specific PRMT7 knockout (EndoKO) mice at 3 months of age. Data are presented as mean ± s.d. ns for not significant. Student’s t-test. **b** Experimental scheme for the induction of PRMT7 ablation in ECs by tamoxifen (Tmx), followed by MI modeling. **c, d** Echocardiographic parameters of left heart hemodynamic alteration at 3 weeks post-MI. AoV VTI: aortic valve velocity time integral. AAT: aorta artery acceleration time. AET: aorta ejection time. **e** Echocardiographic parameters of right heart hemodynamic alteration at 3 weeks post-MI. PV VTI: pulmonary valve velocity time integral. PAT: pulmonary artery acceleration time. PET: pulmonary ejection time (n=3). All data are presented as mean ± s.d. ns for *P* > 0.05, * *P* < 0.05, ** *P* < 0.01, *** *P* < 0.001, **** *P* < 0.0001. One-way ANOVA.


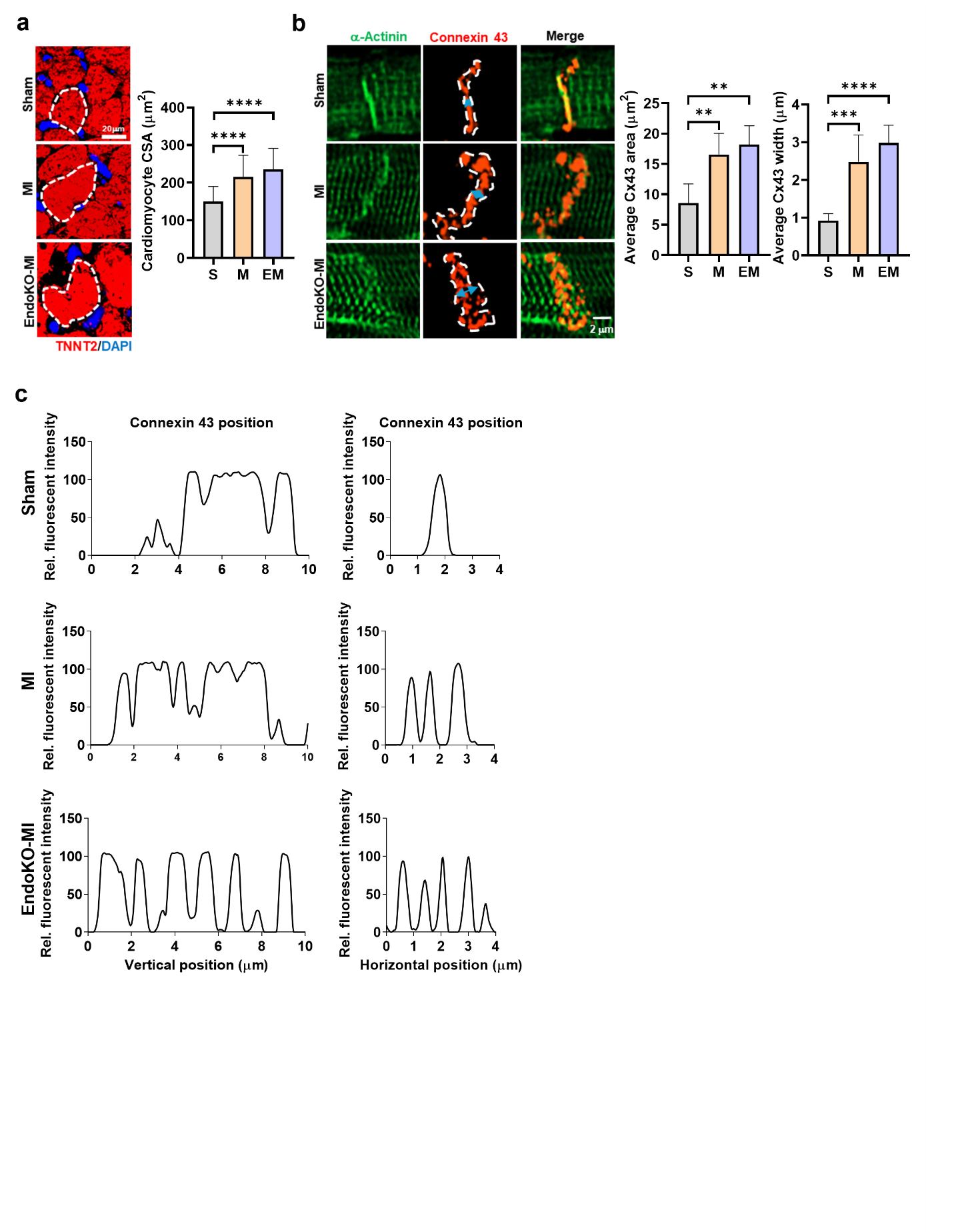


**Supplementary Fig. 4. a** Representative images of heart sections stained for cardiomyocyte (TNNT2, red) and counterstained with DAPI (blue). Scale bar, 20 µm. Quantification of cross- sectional area (CSA) of cardiomyocyte in Sham (S), MI (M), and EndoKO-MI (EM) mice. **b** Representative images of immunostaining for α-Actinin (green) and Connexin 43 (red) in cardiac sections from S, M, and EM mice. Scale bar, 2 µm. Quantification of average Connexin 43 areas and width of heart sections. All data are presented as mean ± s.d. ** *P* < 0.01, **** *P* < 0.0001. One-way ANOVA. **c** Fluorescent intensity of Connexin 43 in vertical and horizontal position from Sham, MI, and EndKO-MI mice.


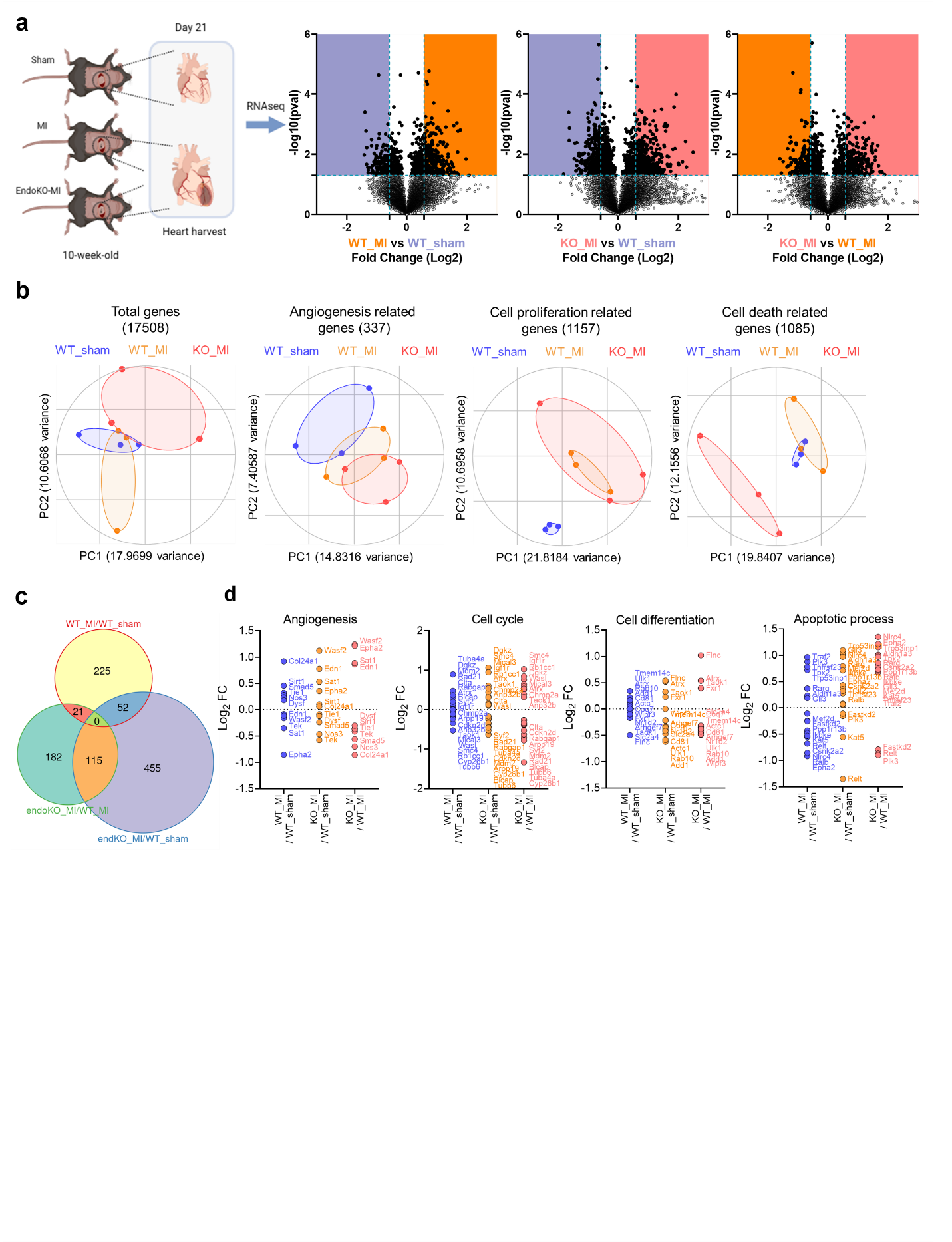


**Supplementary Fig. 5. a** Experimental scheme detailing the RNA-sequencing analysis methodology (left) and volcano plots depicting differential gene expression between experimental groups (right). **b** Principal component analysis (PCA) plots showing the distribution of total genes and genes associated with angiogenesis, cell proliferation, and cell death. **c** Venn diagram of differentially expressed genes between groups. **d** Dot plot displaying specific up-and down-regulated genes related to angiogenesis, cell cycle, cell differentiation, and apoptotic process between groups.
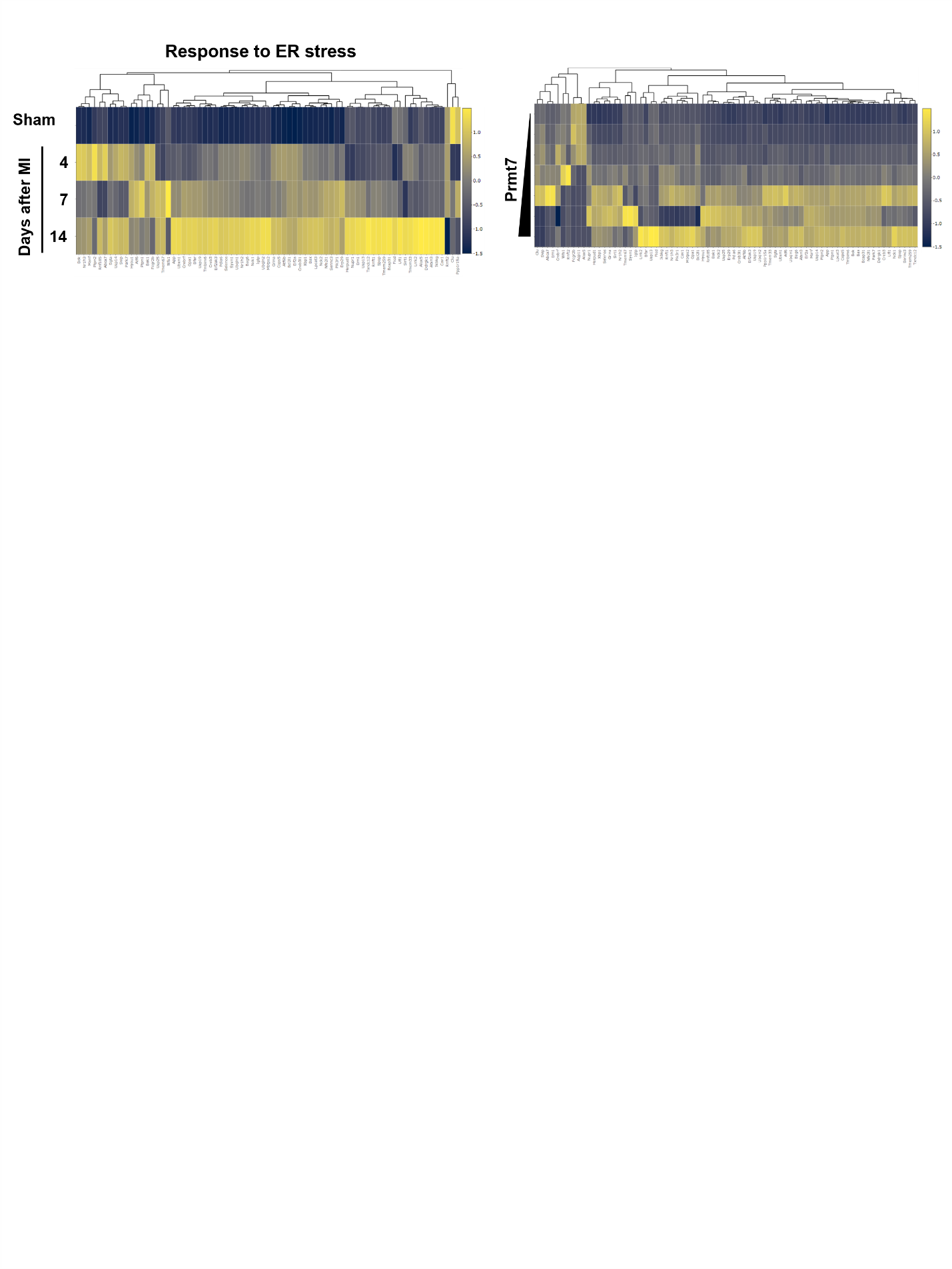


**Supplementary Fig. 6.** Clustered heatmap analysis of differential gene expressions related to endoplasmic reticulum stress response (left) and *Prmt7* expression patterns (right) using murine myocardial infarction dataset (GSE201947) at day 4, 7, and 14.


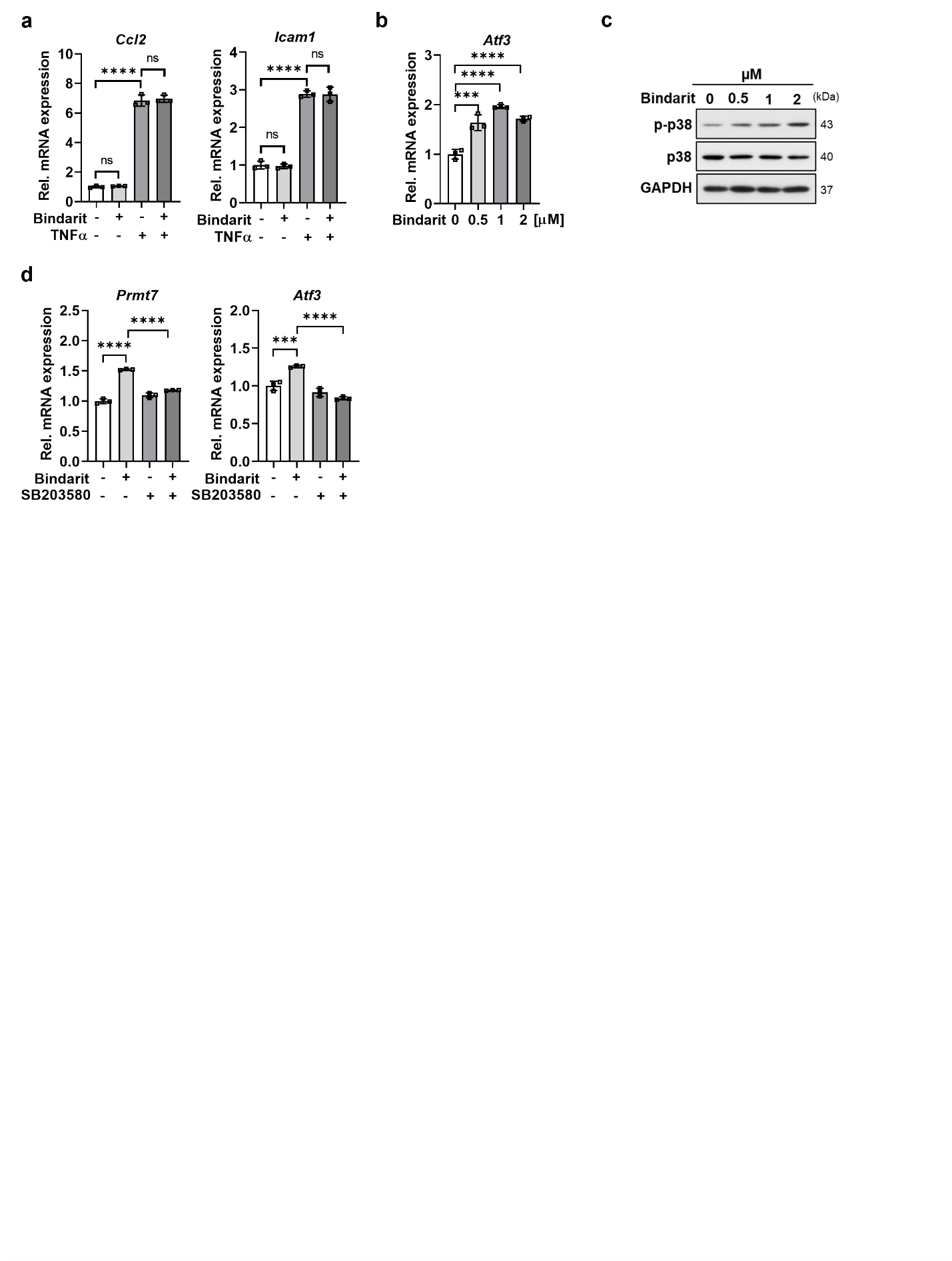


**Supplementary Fig. 7.** **a** Quantitative RT-PCR analysis of *Ccl-2* and *Icam-1* mRNA expressions in C166 cells treated with DMSO, bindarit (500nM), TNF-α (50ng/mL), or a combination of bindarit and TNF-α for 24 hours. **b** Quantitative RT-PCR analysis of *Atf3* mRNA expression in C166 cells treated with DMSO or bindarit at different concentrations of 0.5, 1, or 2µM. **c** Immunoblot analysis of phosphorylated p38 (p-p38), p38, and GAPDH protein levels in C166 cells treated with DMSO or bindarit at different concentrations of 0.5, 1, or 2µM. **d** Quantitative RT-PCR analysis of *Prmt7* and *Atf3* mRNA expressions in C166 cells treated with DMSO, bindarit (500nM), SB203580 (1µM), or a combination of bindarit and SB203580 for 3 hours. All data are presented as mean ± s.d. ns for *P* > 0.05, **** *P* < 0.0001. One-way ANOVA.


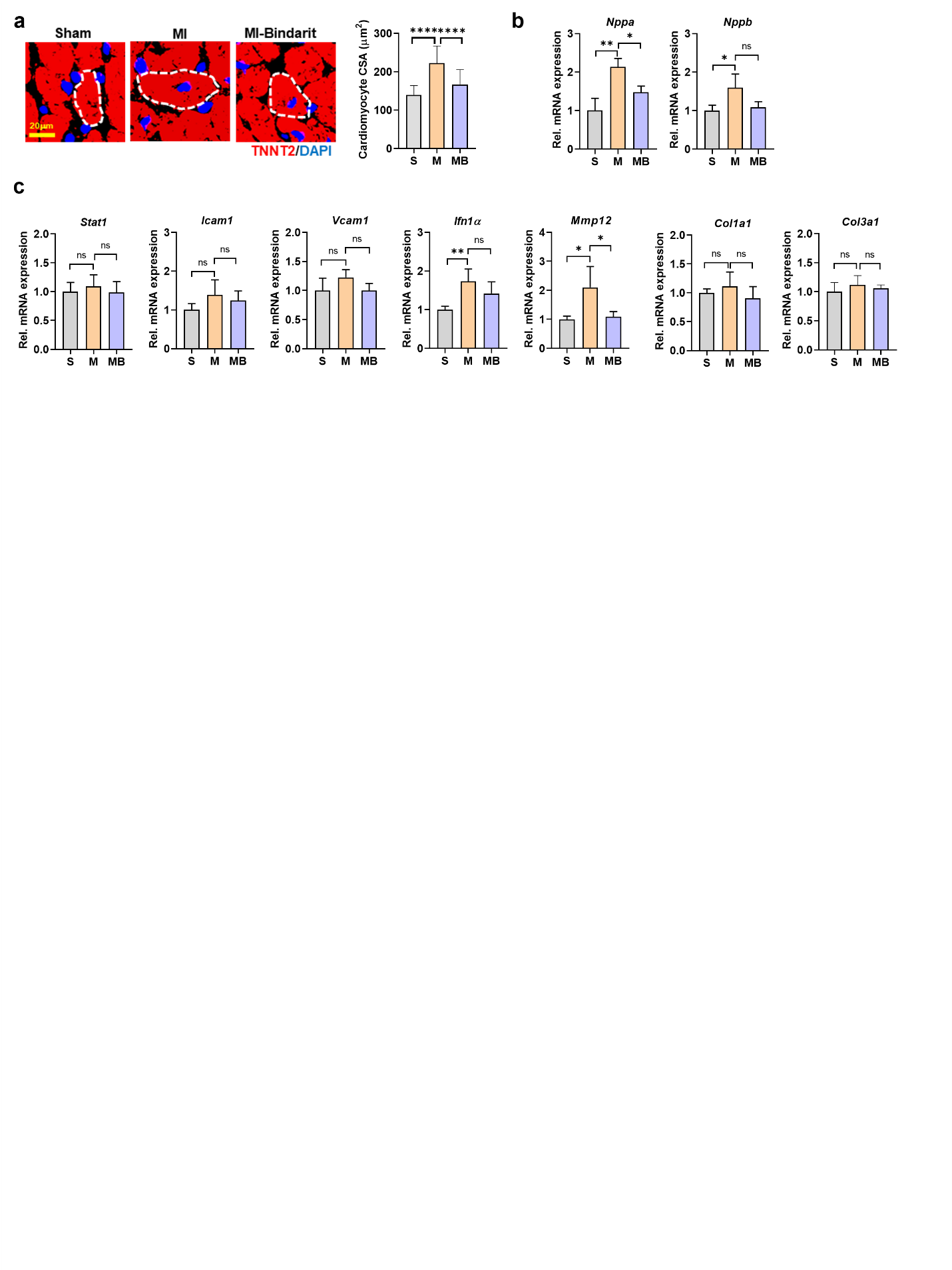


**Supplementary Fig. 8. a** Representative image of heart tissue stained for cardiomyocyte (TNNT2, red) and counterstained with DAPI (blue). Scale bar, 20 µm. Quantification of cross-sectional area (CSA) of cardiomyocyte in Sham (S), MI (M), and MI-Bindarit (MB) mice. **b** Quantitative RT-PCR analysis of *Nppa* and *Nppb* mRNA expression of heart tissues (n=3). **c** Quantitative RT-PCR analysis of *Stat1*, *Icam1*, *Vcam1*, *Ifn1α*, *Mmp12*, *Col1a1*, *Col3a1* mRNA expression of liver tissues (n=3). All data are presented as mean ± s.d. *** *P* < 0.001, **** *P* < 0.0001. One-way ANOVA.


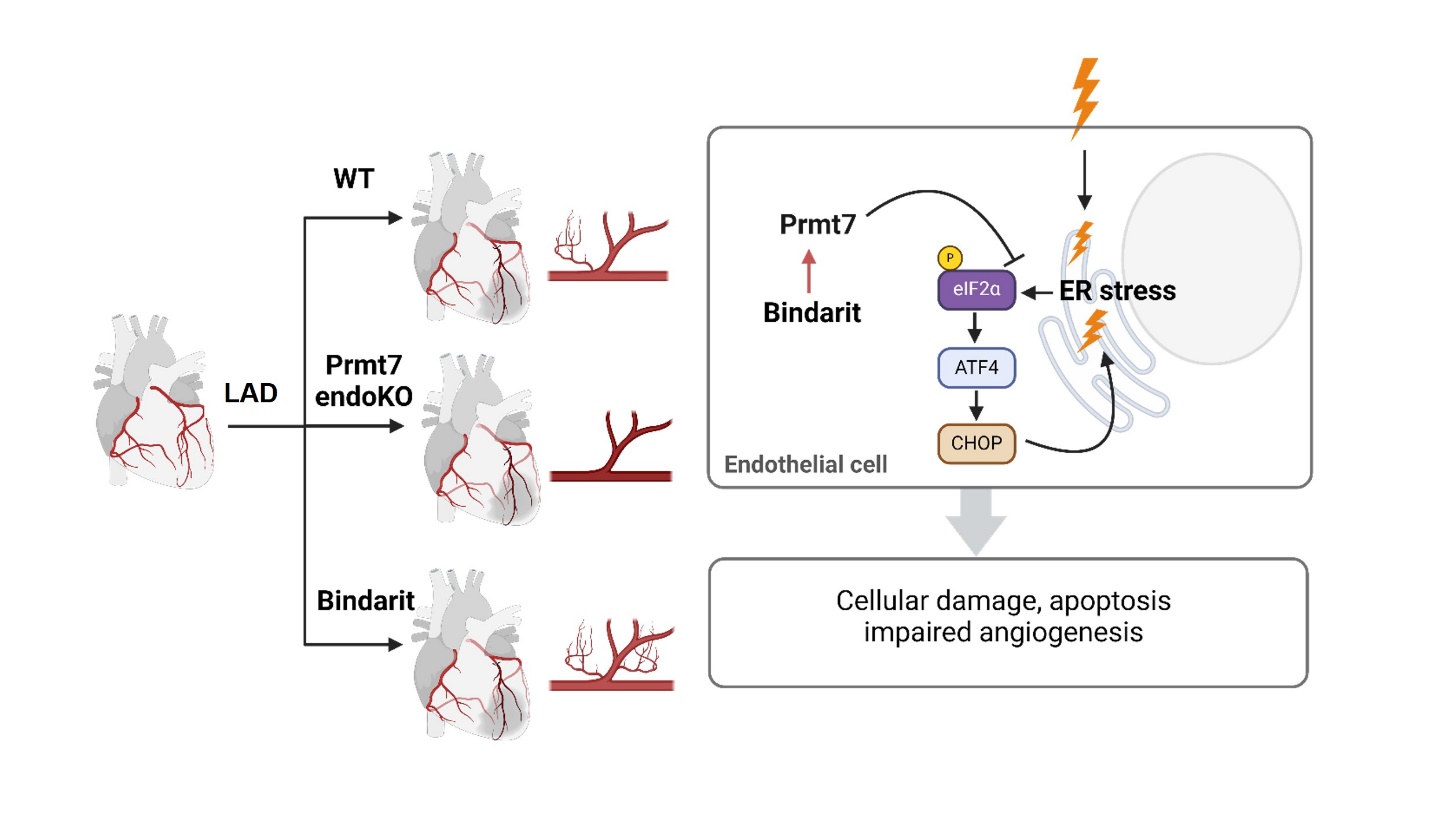


**Supplementary Fig. 9.** Working model of PRMT7 and PRMT7-inducer bindarit in myocardial infarction.

Supplementary Table 1. List of primary antibodies used in this study.

| Antigen | Cat No. Manufacturer | |
| --- | --- | --- |
| GAPDH | LF-PA0018 | AbFrontier |
| PRMT7 | Sc98882 | Santa Cruz |
| α-Actinin | A7732 | Sigma Aldrich |
| p-eIF2α | 3398S | Cell Signaling |
| eIF2α | 5324S | Cell Signaling |
| p53 | sc-126 | Santa Cruz |
| Bax | Sc-7480 | Santa Cruz |
| p-p38 | 9211 | Cell Signaling |
| p38 | 9212 | Cell Signaling |
| Collagen type 1 | Ab260043 | Abcam |
| γH2AX | NB100-384 | Novus |
| Connexin43 | 3512S | Cell Signaling |
| BrdU | sc-32323 | Santa Cruz |
| CDH5 | MABT886 | Sigma (Millipore) |
| CHOP | 2895S | Cell Signaling |
| ATF4 | 11815S | Cell Signaling |
| TNNT2 | Abcam | Ab10214 |
| VEGFR2 | Cell Signaling | 2479S |
| HA-Tag | Cell Signaling | 3724S |

Supplementary Table 2. List of primer sequences used for quantitative RT-PCR analysis in this study.

| Gene symbol |  | Sequences |
| --- | --- | --- |
| *Prmt7* | Forward | 5'-TTC-CCA-CAG-CGG-GCA-TTA-T-3' |
|  | Reverse | 5'-TGT-AGC-ATG-TCG-GCA-TAG-GA-3' |
| *MKi67* | Forward | 5'-TCA-TGA-GGA-TGG-AAG-CAA-GCC-3' |
|  | Reverse | 5'-CTC-ACT-CTT-CTC-AGG-GTC-AGC-A-3' |
| *Icam1* | Forward | 5'-AAC-TGT-GGC-ACC-GTG-CAG-TC-3' |
|  | Reverse | 5'-AGG-GTG-AGG-TCC-TTG-CCT-ACT-TG-3' |
| *Gapdh* | Forward | 5’-GAC-ATG-CCG-CCT-GGA-GAA-AC-3’ |
|  | Reverse | 5’-AGC-CCA-GGA-TGC-CCT-TTA-GT-3’ |
| *Tnfα* | Forward | 5'- GGT-GCC-TAT-GTC-TCA-GCC-TCT-T -3' |
|  | Reverse | 5'- GCC-ATA-GAA-CTG-ATG-AGA-GGG-AG -3' |
| *Il1α* | Forward | 5'-GGA-GAA-GAC-CAG-CCC-GTG-TTG-CT-3' |
|  | Reverse | 5'-CCG-TGC-CAG-GTG-CAC-CCG-ACT-T-3' |
| *Bax* | Forward | 5‘-AAA-CTG-GTG-CTC-AAG-GCC-C-3’ |
|  | Reverse | 5‘-GGT-CCC-GAA-GTA-GGA-GAG-GA-3’ |
| *p53* | Forward | 5’-GGG-GAG-GAG-CCA-GGC-CAT-CA-3’ |
|  | Reverse | 5’-CCG-CGC-CAT-GGC-CAT-CTA-CA-3’ |
| *Bnip3* | Forward | 5’-GCT-CCA-AGA-GTT-CTC-ACT-GTG-AC-3’ |
|  | Reverse | 5’-GTT-TTT-CTC-GCC-AAA-GCT-GTG-GC-3’ |
| *Bcl2* | Forward | 5’-CCT-GTG-GAT-GAC-TGA-GTA-CCT-G-3’ |
|  | Reverse | 5’-AGC-CAG-GAG-AAA-TCA-AAC-AGA-GG-3’ |
| *Chop* | Forward | 5’-TAT-CTC-ATC-CCC-AGG-AAA-CG-3’ |
|  | Reverse | 5’-CAG-GGT-CAA-GAG-TAG-TGA-AGG-TTT-3’ |
| *Atf4* | Forward | 5’-AAC-CTC-ATG-GGT-TCT-CCA-GCG-A-3’ |
|  | Reverse | 5’-CTC-CAA-CAT-CCA-ATC-TGT-CCC-G-3’ |
| *Ccl2* | Forward | 5’-GCT-ACA-AGA-GGA-TCA-CCA-GCA-G-3’ |
|  | Reverse | 5’-GTC-TGG-ACC-CAT-TCC-TTC-TTG-G-3’ |
| *Vegfa* | Forward | 5‘- CTG-CTG-TAA-CGA-TGA-AGC-CCT-G -3’ |
|  | Reverse | 5‘- GCT-GTA-GGA-AGC-TCA-TCT-CTC-C -3’ |
| *Vegfr2* | Forward | 5’-CGA-GAC-CAT-TGA-AGT-GAC-TTG-CC-3’ |
|  | Reverse | 5’-TTC-CTC-ACC-CTG-CGG-ATA-GTC-A-3’ |
| *Atf3* | Forward | 5’-GAA-GAT-GAG-AGG-AAA-AGG-AGG-CG-3’ |
|  | Reverse | 5’-GCT-CAG-CAT-TCA-CAC-TCT-CCA-G-3’ |
| *Nppa* | Forward | 5’-GCT-TCC-AGG-CCA-TAT-TGG-AG-3’ |
|  | Reverse | 5’-GGG-GGC-ATG-ACC-TCA-TCT-T-3’ |
| *Nppb* | Forward | 5’-TCC-TAG-CCA-GTC-TCC-AGA-GCA-A-3’ |
|  | Reverse | 5’-GGT-CCT-TCA-AGA-GCT-GTC-TCT-G-3’ |
| *Stat1* | Forward | 5’-GCC-TCT-CAT-TGT-CAC-CGA-AGA-AC-3’ |
|  | Reverse | 5’-TGG-CTG-ACG-TTG-GAG-ATC-ACC-A-3’ |
| *Vcam1* | Forward | 5’-GCC-ACC-CTC-ACC-TTA-ATT-GCT-ATG-3’ |
|  | Reverse | 5’-TGT-GCA-GCC-ACC-TGA-GAT-CC-3’ |
| *Ifn1α* | Forward | 5’-GGA-TGT-GAC-CTT-CCT-CAG-ACT-C-3’ |
|  | Reverse | 5’-ACC-TTC-TCC-TGC-GGG-AAT-CCA-A-3’ |
| *Mmp12* | Forward | 5’-CAC-ACT-TCC-CAG-GAA-TCA-AGC-C-3’ |
|  | Reverse | 5’-TTT-GGT-GAC-ACG-ACG-GAA-CAG-G-3’ |
| *Col1a1* | Forward | 5’-TCA-TCG-TGG-CTT-CTC-TGG-TC-3’ |
|  | Reverse | 5’-GAC-CGT-TGA-GTC-CGT-CTT-TG-3’ |
| *Col3a1* | Forward | 5’-GAC-CAA-AAG-GTG-ATG-CTG-GAC-AG-3’ |
|  | Reverse | 5’-CAA-GAC-CTC-GTG-CTC-CAG-TTA-G-3’ |
